# Ligand-assisted facile Co^2+^/Co^3+^ conversion in a metal–hydroxide framework promotes electrochemical benzyl alcohol oxidation at ampere-scale current density

**DOI:** 10.1039/d6sc06869c

**Published:** 2026-10-29

**Authors:** Toufik Ansari, Harshit Gupta, Manshu Dhillon, Aviru Kumar Basu, Arindam Indra

**Affiliations:** a Department of Chemistry, IIT (BHU) Varanasi UP-221005 India arindam.chy@iitbhu.ac.in; b Institute of Nano Science and Technology Knowledge City, Sector 81 Mohali Punjab 140306 India

## Abstract

The electrooxidation of benzyl alcohol at the anode has emerged as an alternative to the kinetically sluggish oxygen evolution reaction (OER). Although cobalt-based electrocatalysts have been widely explored for the OER, their catalytic performance for anodic alcohol oxidation reactions remains limited due to the substantial energy barrier arising from the transformation of the precatalyst to the active Co(O)OH phase and its weak interaction with benzyl alcohol. Herein, a ligand-modification strategy is adopted by incorporating chloranilic acid as a redox-active functional ligand into the structure of FeCo-layered double hydroxide (LDH) to form a metal hydroxide organic framework (HOF). The quinonoid structure of chloranilic acid in the HOF facilitates the electrooxidation of Co^2+^ to Co^3+^ to form Fe–Co^3+^(O)OH as the active phase. As a result, FeCo-HOF-AC produces higher benzyl alcohol oxidation activity, suppressing the OER, compared to the active catalyst FeCo-LDH-AC. Interestingly, FeCo-HOF-AC achieved 1.6 A cm^−2^ current density for benzyl alcohol oxidation at 1.39 V_RHE_ at an elevated temperature (65 °C), with 98% faradaic efficiency for PhCOOH.

## Abstract 2

Redox active ligand chloranilic acid-assisted structural modification of FeCo-layered double hydroxide has been described to access a Co^3+^-rich Fe–Co(O)OH active phase for improved benzyl alcohol oxidation with 98% faradaic efficiency.

## Graphical abstract

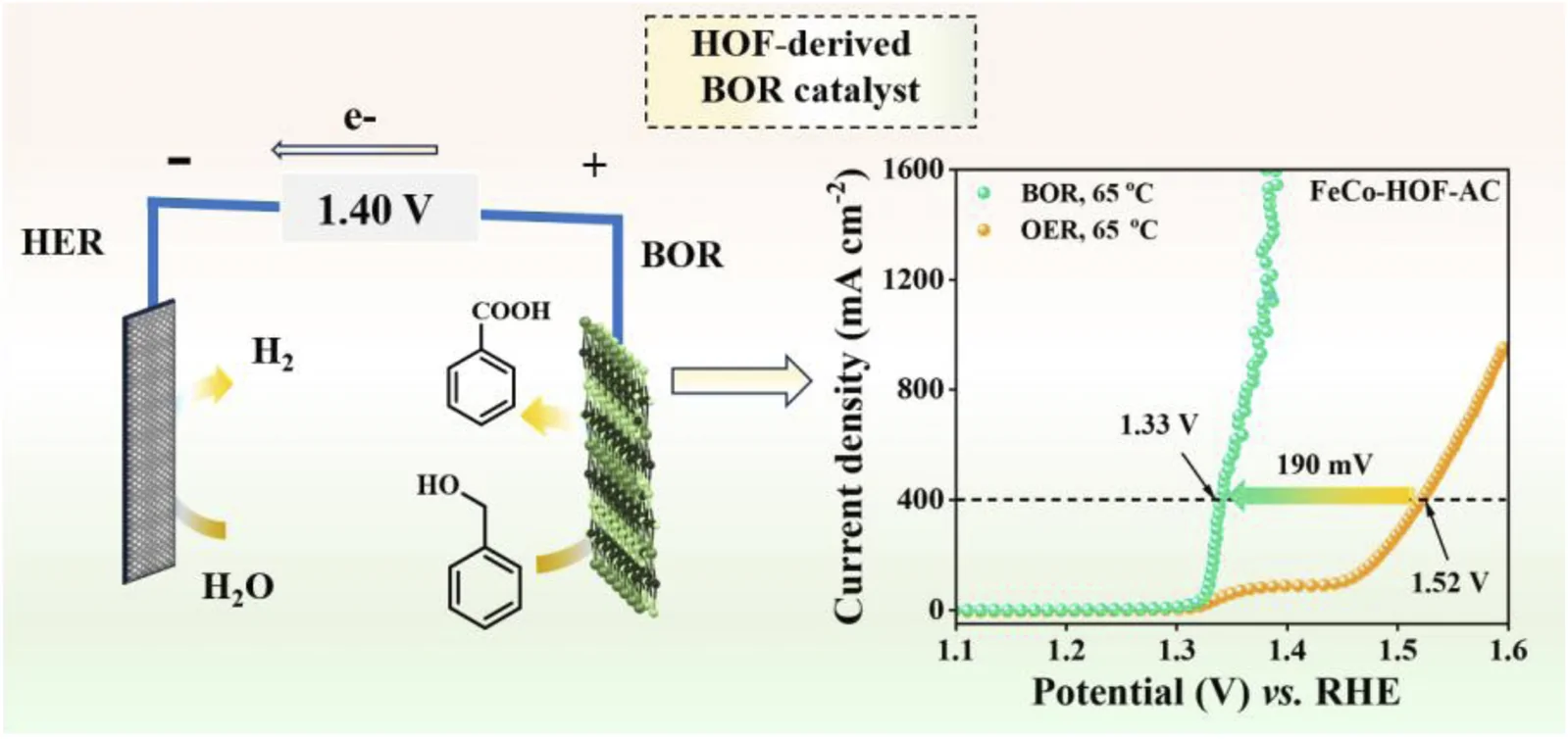

## Introduction

The conversion of renewable carbon resources into valuable products is a key factor in sustainable chemical synthesis. In this context, benzyl alcohol, a biomass-derived substrate, can be oxidized to PhCOOH, commonly utilised in chemical industries.^1,2^ Although the conventional industrial process of benzoic acid production deals with the stoichiometric oxidation of toluene with oxidizing agents like CrO_3_, KMnO_4_, *etc.*, the recent developments in thermocatalytic oxidation of benzyl alcohol to benzoic acid are noteworthy.^1,2^ However, these processes cannot be effectively translated into the industrial scale because of the complexity of specially designed molecular complexes with expensive ligands, the use of different oxidizing agents and additives, and the difficulties in the activation of molecular oxygen as the terminal oxidant. As a result, a high temperature is often required to activate molecular O_2_ and carry out the oxidation of PhCH_2_OH to PhCOOH by a thermocatalytic process.^2^ Furthermore, the photocatalytic approaches have been developed where the use of expensive photosensitizers/semiconductors and molecular complexes limits their large-scale applications.^2^

Consequently, the development of alternative and more sustainable approaches for the selective synthesis of PhCOOH is highly desirable. In this context, the electrocatalytic benzyl alcohol oxidation reaction has emerged as an attractive alternative to conventional thermocatalytic and photocatalytic oxidation processes.^3^ Electrochemical oxidation offers several potential advantages, including high purity of product, easy product separation and purification, and catalyst recycling.^3^ However, the electrocatalytic benzyl alcohol oxidation reaction (BOR) in the aqueous medium faces several challenges like low current density, interference from the oxygen evolution reaction (OER), and high potential requirements. Although the thermodynamic potential of PhCH_2_OH oxidation to PhCOOH (0.48 V) is much lower than that of water oxidation (1.23 V), the BOR requires >2.00 V to attain an industrially relevant current density (400 mA cm^−2^).^3,4^ At such a high potential, often interference from the OER is observed, reducing the faradaic efficiency (FE) of PhCOOH production. Therefore, catalyst design for the BOR requires special attention with a detailed understanding of the reaction mechanism.^5^

The transition metal-derived metal (oxy)hydroxide, M(O)OH, produced by the anodic activation of different precatalysts, such as layered double hydroxides (LDHs), metal oxides, chalcogenides, phosphides, metal–organic frameworks (MOFs), *etc.*, produces reasonable PhCH_2_OH oxidation activity in a strongly alkaline medium.^6^ However, most of the reported catalysts require relatively high applied potentials and operate at room temperature, restricting the realistic current density and FE. Among the different transition-metal-based catalysts, Co(O)OH is considered a promising one for the BOR due to the dynamic redox chemistry associated with the Co^2+/3+/4+^ ions.^7^ The electrooxidation of Co-based catalysts primarily involves two redox couples: (i) the Co^2+^/Co^3+^ couple at lower applied electrode potentials (1.0–1.3 V_RHE_) and (ii) the Co^3+^/Co^4+^ couple at higher potentials (1.3–1.5 V_RHE_). It is generally accepted that Co(O)OH with Co^3+^-rich species drives PhCH_2_OH oxidation, whereas Co^IV^=O preferentially promotes the competing OER.^8,9^

Several strategies, such as hetero-metal doping, selective metal leaching, vacancy formation, defect engineering, and anion regulation, have been explored to promote the oxidation of Co^2+^ to Co^3+^ and the formation of the Co^3+^(O)OH active phase during electrochemical alcohol oxidation.^9^

For example, Li *et al.* reported a significant improvement in the anodic oxidation of PhCH_2_OH with Au/Co(O)OH compared to Co(O)OH due to enhanced adsorption of benzyl alcohol at the interface of the two materials.^11^ The Baltrusaitis group explored the electronic effects of Ni and Mn incorporation into Co(O)OH.^12^ While the Ni incorporation into Co(O)OH was found to promote the formation of catalytically active Co^3+^ species, the Mn incorporation into the same catalyst suppressed the active site formation, thereby reducing the BOR activity.

Furthermore, amorphous Ni–Co hydroxide nanosheets were demonstrated to achieve synergistic interaction between Ni and Co sites for an improved BOR activity.^13^ Recently, we also reported Co^3+^-rich Co(O)OH, attained by the selective leaching of Cr ions during the anodic activation of the precatalyst, to optimize the substrate adsorption on the catalyst surface and accelerate PhCH_2_OH oxidation.^14^

However, to date, only a few catalysts have been demonstrated to operate at high temperature (>60 °C) and achieve an industrial current density. The main challenge is associated with the competition from the OER to significantly reduce the FE, especially at the applied anodic potential > 1.4 V_RHE_. Therefore, it is essential to develop efficient catalysts capable of functioning at elevated temperatures and delivering industrial current densities at a lower potential value, preferentially < 1.4 V_RHE_. For example, Ghosh *et al.* demonstrated the use of self-supported Ni_3_N/NM (NM = nickel mesh) for benzyl alcohol oxidation at an elevated temperature.^3^ Furthermore, Hausmann *et al.* reported the use of plasma-treated nickel foam (NF) to achieve an industrially relevant current density at 65 °C.^15^

In this context, Co^3+^(O)OH-based catalyst systems for the BOR have never been explored at an elevated temperature. Herein, we have demonstrated the performance of Fe–Co(O)OHs, derived from two precatalysts, FeCo-HOF (a metal–hydroxide framework) and FeCo-LDH, for the BOR to achieve an industrial-scale current density at a moderately high temperature (65 °C) (Scheme 1).

**Scheme 1:**
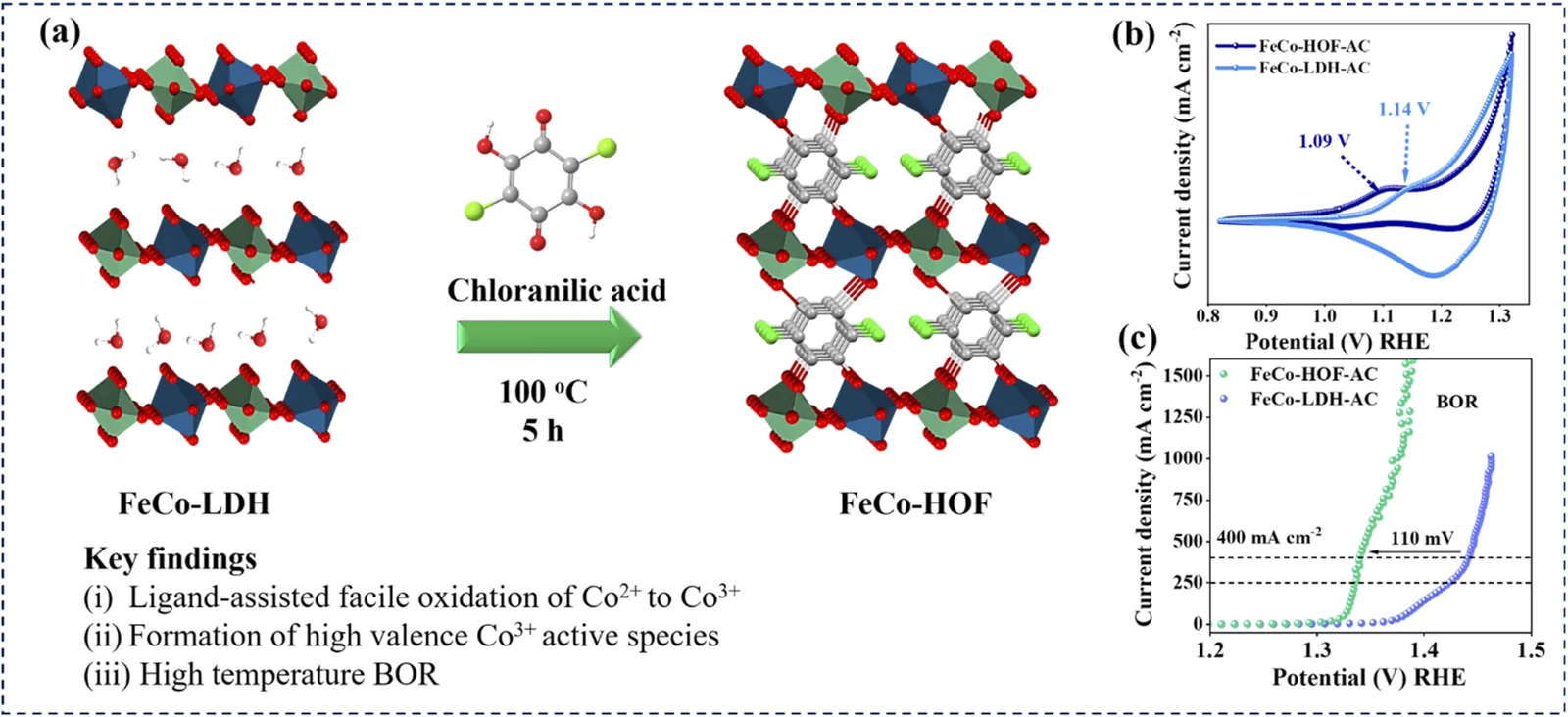
(a) The schematic representation showing the formation of the hydroxide organic framework from layered double hydroxide. (b) CV profiles demonstrating facile Co^2+^/Co^3+^ oxidation in FeCo-HOF compared with that in the corresponding LDH. (c) LSV curves showing the superior BOR activity of FeCo-HOF-AC over FeCo-LDH at 65 °C.

The regulation of the coordination environment of metal centres in HOFs through an organic ligand-modification strategy optimizes the electronic structure by electron redistribution.^16–19^ Several research groups have reported the coordination of carboxylic acid ligands to metal ions in HOFs for an improved electrocatalytic activity.^20–24^

For example, the coordination of glutamic acid to the metal ions of Ni(OH)_2_ altered the Ni–O bond lengths and coordination number, and the oxidation state of Ni to improve the OER activity.^20^ While the oxalate-modified CoNi-hydroxide improved the glycerol oxidation,^21^ terephthalic acid^22^ and benzoic acid modification of the same catalyst facilitated 5-hydroxymethylfurfural (HMF) oxidation.^8^ Recently, the Shi group reported the introduction of furan dicarboxylic acid, formed by the anodic oxidation of HMF, into a Ni(OH)_2_ framework for an improved catalytic activity.^23^ This ligand modification induces strong electronic interactions, facilitates proton dissociation and proton-coupled electron transfer (PCET), and promotes the formation of catalytically active species to improve the performance of the metal hydroxide-based electrocatalysts.

In all the above cases, the coordination of the redox-innocent ligand (carboxylic acid) to the metal ions helps in the redistribution of the electron over the metal centre and the ligand to alter the electronic structure.^8,23^ In contrast, a redox-active ligand like quinol/quinone can effectively take part in the redox process during the anodic reaction and redistribute the charges over the metal ions and ligand to make the catalytic process more efficient.^24^ However, the modification of the metal hydroxide with a non-innocent ligand for an improved electrocatalytic activity is rarely studied.

The lacunae in this field motivated us to choose a non-innocent ligand, chloranilic acid, to modify the FeCo-LDH framework for an improved electrochemical benzyl alcohol oxidation activity. The redox-active chloranilic acid with a quinonoid structure modulates the Co^2+^/Co^3+^ redox couple, promotes synergistic interactions between the active metal sites, and improves the electrochemical activity.^7–9^

The facile Co^2+^ to Co^3+^ oxidation in FeCo-HOF promotes the formation of the active catalyst Fe–Co(O)OH (FeCo-HOF-AC) with abundant high-valent Co^3+^ species, responsible for the selective oxidation of benzyl alcohol. As a result, FeCo-HOF-AC achieved an industrial-scale current density of 400 mA cm^−2^ (at 65 °C) at a very low electrode potential of 1.33 V_RHE_, superior to FeCo-LDH-AC and other literature-reported catalyst systems. The complete conversion of PhCH_2_OH to PhCOOH was attained with ≥98% FE of the product.

Furthermore, when FeCo-HOF-AC was used as the anodic catalyst in a two-electrode electrolyzer, a cell voltage of only 1.71 V was required to achieve a current density of 400 mA cm^−2^, which is substantially lower than the OER overpotential (1.99 V). This significant reduction in cell voltage highlights the superior energy efficiency of the overall PhCH_2_OH oxidation-assisted electrolyzer.

## Results and discussion

### Characterisation of the precatalysts

First, FeCo-LDH was deposited on NF by a hydrothermal method and treated with chloranilic acid to produce FeCo-HOF (Scheme 1 and Table S1). For the comparison purpose, Co-HOF was also produced from Co-LDH. The use of redox-active chloranilic acid as the organic linker redistributes the charge density in FeCo-HOF compared to that in FeCo-LDH (see later).^24^ The increased electrical conductivity of chloranilate frameworks was also demonstrated in earlier reports.^24–26^

The formation of FeCo-LDH and FeCo-HOF was confirmed by powder X-ray diffraction (PXRD) studies (Fig. S1).^6,24^ The PXRD revealed a negative shift (0.24° in the two theta value) of the (001) face in FeCo-HOF, compared to that in the corresponding LDH, suggesting an expansion (0.017 nm) of the inter-layer distance of the metal hydroxide layers due to ligand incorporation. The Fourier-transform infrared (FTIR) spectra showed the stretching vibrations of the C=O group at 1643 cm^−1^, a slightly lower wavenumber compared to that of free chloranilic acid (Fig. S2).^24–26^

Furthermore, the Raman study of FeCo-LDH exhibited two characteristic peaks at 468 cm^−1^ and 530 cm^−1^ for Co–O bending (E_g_) and Co–O stretching (A_1g_) modes, respectively.^27^ Similarly, FeCo-HOF also showed two prominent bands corresponding to E_g_ (400 cm^−1^) and A_1g_ (590 cm^−1^) modes of Co–O (Fig. 1a).^28^ The appearance of A_1g_ modes at a higher Raman shift value in FeCo-HOF suggests that the ligand coordination in the HOF modifies the electronic configuration, leading to enhanced covalency around the Co centers.

**Fig. 1:**
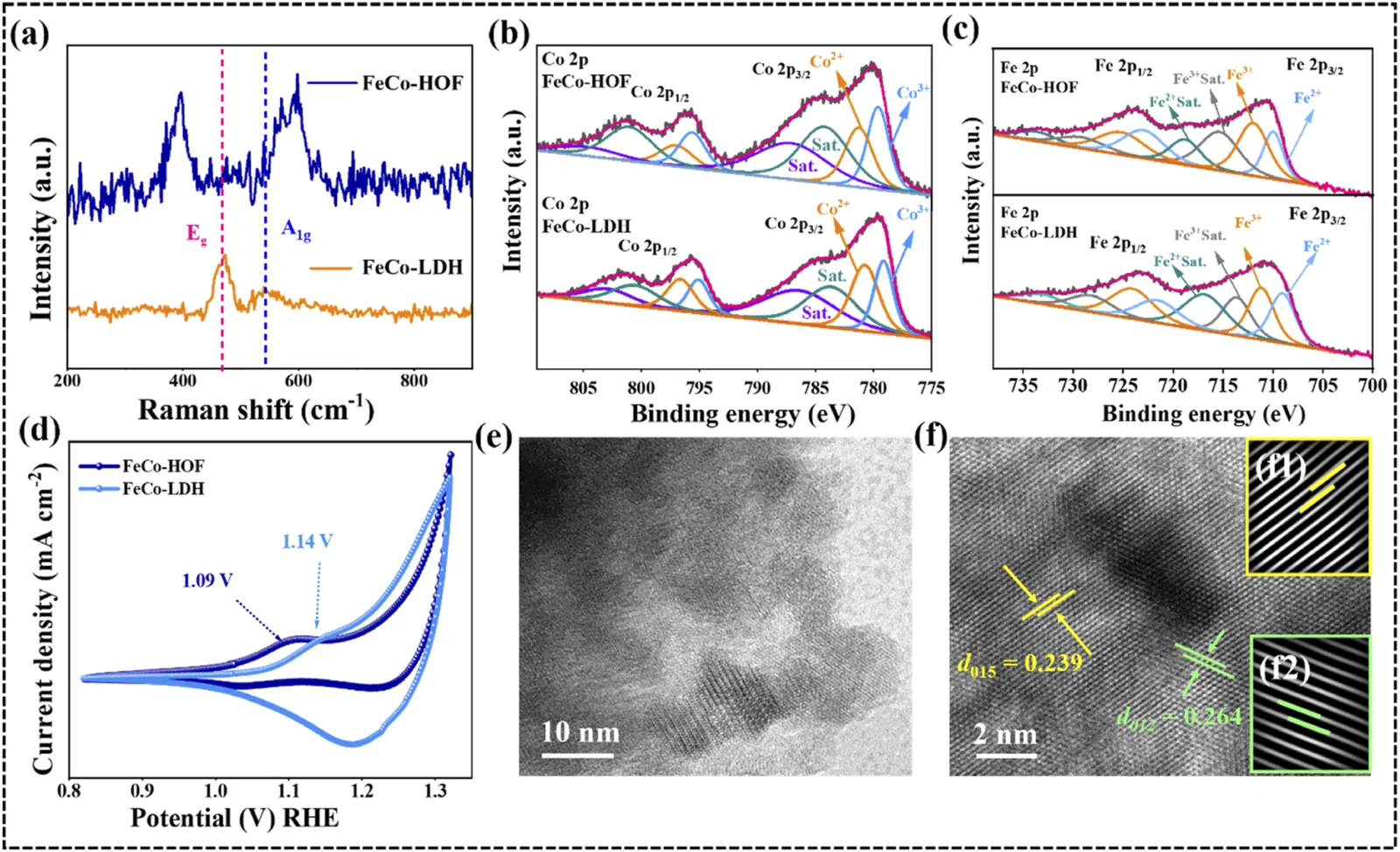
(a) Raman spectrum of FeCo-HOF and FeCo-LDH, showing Co–O bending (E_g_) and Co–O stretching (A_1g_) vibrations. (b) Comparison of the Co 2p XPS of FeCo-HOF and FeCo-LDH. (c) Comparison of the Fe 2p XPS of FeCo-HOF and FeCo-LDH. (d) Comparison of the CV profiles of FeCo-HOF and FeCo-LDH showing the facile oxidation of Co^2+^/Co^3+^ in the ligand-coordinated hydroxide framework structure. (e and f) HR-TEM images of FeCo-HOF detected the (015) and (012) planes of Co(OH)_2_. (f1 and f2) The inverse FFT images of the specified area in figure (f), confirming the crystalline structure with the (015) and (101) planes of Co(OH)_2._

The X-ray photoelectron spectroscopy (XPS) also revealed the modification of the electronic structure of FeCo-LDH after the ligand coordination.^6,27^ The Co 2p XPS of FeCo-HOF showed a positive shift in the binding energy of both Co 2p_3/2_ and Co 2p_1/2_ peaks compared to that of FeCo-LDH (Fig. 1b).^6^ The electron redistribution after the ligand coordination to the metal center results in a more positive charge density on the cobalt ions, as reflected by Co 2p XPS. A similar conclusion can be drawn from the Co^3+^/Co^2+^ peak area ratio, which shows a higher value for FeCo-HOF (1.12) than that for FeCo-LDH (0.87) (Fig. 1b).^29,30^

The Fe 2p XPS of FeCo-HOF also showed the shift of the Fe 2p_3/2_ and Fe 2p_1/2_ peaks towards the higher binding energies compared to that of FeCo-LDH, suggesting a higher amount of high-valence Fe^3+^ in the HOF (Fig. 1c).^24,31^

The O 1s XPS of FeCo-LDH and FeCo-HOF were deconvoluted into lattice oxygen (O_1_), –OH groups (O_2_), and adsorbed H_2_O molecules (O_3_) (Fig. S3).^31,32^ Compared to FeCo-LDH, in FeCo-HOF, a notable negative shift of the O_1_ and O_2_ peaks was observed, suggesting electron transfer from the metal centres to the ligand framework. This observation directly supports the strong metal–oxygen interaction in FeCo-HOF.

The C 1s-XPS of FeCo-HOF and FeCo-LDH showed three peaks for C–C, C–O, and C=O species (Fig. S4).^32–34^

The XPS reveals the formation of electron-deficient Co and Fe centres in FeCo-HOF-AC, consistent with the Raman analysis. The strong electron-withdrawing nature of chloranilic acid results in the transfer of the electron density from the Fe/Co centres to the ligand framework, leaving the metal sites electron-deficient. Such electron-deficient Co/Fe centres facilitate the generation of high-valence metal species under anodic conditions, which are recognised as the catalytically active sites for PhCH_2_OH oxidation.

The cyclic voltammetry (CV) study further revealed the Co^2+^/Co^3+^ oxidation at a lower potential value (1.09 V_RHE_) in FeCo-HOF than that in FeCo-LDH-AC (1.14 V_RHE_) (Fig. 1d and S5).^9^ Similarly, Co–HOF shows the Co^2+^/Co^3+^ oxidation peak at a lower potential value than that of Co-LDH (Fig. S5). This clearly indicates that the quinonoid structure of chloranilic acid in the HOFs facilitates the oxidation of Co^2+^ to Co^3+^, forming Co^3+^(O)OH as the active phase. This behavior suggests that the modified electronic structure and improved conductivity introduced by the HOF framework effectively accelerate the formation of active species Co^3+^(O)OH, thereby contributing to the catalytic performance.

Although ligand coordination in the HOF facilitates the oxidation of Co^2+^ to Co^3+^, the incorporation of Fe further promotes this redox transformation by modifying the local electronic environment of the Co centres. Interestingly, the CV profile of the hydrothermally synthesised β-CoOOH exhibits a Co^3+^/Co^2+^ redox peak comparable to that of HOF-derived FeCo(O)OH but with a poor catalytic activity (see later). Furthermore, the CV profile of the free chloranilic acid ligand did not show any redox peaks in the studied potential region, indicating that the ligand does not contribute to the observed CV response (Fig. S5).

The morphology of the precatalysts was examined using scanning electron microscopy (SEM) (Fig. S6). The SEM images revealed that both FeCo-LDH and FeCo-HOF have flower-like morphologies, but with significantly different structural features (Fig. S6). Energy-dispersive X-ray (EDX) spectroscopy further confirmed the elements Fe, Co, C, and O within the HOF and LDH frameworks (Fig. S7 and S8).

Transmission electron microscopy (TEM) detected the nanosheet structure of FeCo-HOF. The high-resolution TEM (HRTEM) and corresponding inverse fast Fourier transform (FFT) images revealed *d*-spacings of 0.239 nm and 0.264 nm, corresponding to the (015) and (012) planes of FeCo-HOF, respectively (Fig. 1e and f).^35,36^

### Electrochemical reconstruction of the precatalysts

The electrochemical reconstruction of the precatalysts into active catalysts was conducted in 1.0 M KOH solution using cyclic voltammetry (CV). The electrochemical activation of FeCo-HOF required 45 CV cycles to produce FeCo(O)OHs (FeCo-HOF-AC) as the active phase. In contrast, FeCo-LDH is transformed into FeCo-LDH-AC only after 25 CV cycles (Fig. S9). The longer activation period required for FeCo-HOF can be attributed to the comparatively robust coordination framework of the HOF with a stronger Co–O bond than the corresponding LDH. Consequently, a larger number of CV cycles is required to achieve ligand dissociation and hydroxylation to generate the catalytically active FeCo(O)OH phase compared to FeCo-LDH.

After the electrochemical reconstruction, IR spectroscopy revealed a complete disappearance of the characteristic C=O (1600–1650 cm^−1^) stretching frequency in the HOF (Fig. S10).^24^

The double-layer capacitance (*C*_dl_) was measured to determine the electrochemically active surface area (ECSA). FeCo-HOF-AC exhibited a higher *C*_dl_ value (10.11 mF dec^−1^), indicating a larger electrochemically active surface area than that of the corresponding LDH (Fig. S11 and Table S2). The higher ECSA confirms that the structural modulation of LDH by Fe incorporation and chloranilic acid offers a higher number of accessible catalytically active sites.

Additionally, the charge transfer properties of the catalysts were assessed through electrochemical impedance spectroscopy (EIS) (Fig. S12 and Table S2). The EIS of FeCo-HOF-AC showed a smaller charge transfer resistance compared to LDH, showing the improvement in the electronic conductivity after the ligand and Fe^3+^ modification.

Furthermore, the redox peak area integration approach was used to assess the number of active sites (Fig. S13).^37^ The FeCo-HOF-AC showed a larger number of active sites (1.26 × 1018) compared to the LDH.

### Characterization of active catalysts

The PXRD patterns of FeCo-HOF-AC and FeCo-LDH-AC matched well with those of a mixed phase of β-Co(O)OH (JCPDF no-06-0075) and α-Co(OH)_2_ (JCPDF no-38-0175) (Fig. S14).^14^ All diffraction peaks of β-Co(O)OH can be indexed to a hexagonal unit cell containing three CoO_2_ layers, consistent with the space group: *R*3*m* (*a* = *b* = 2.84 Å and *c* = 13.16 Å). Co(O)OH exists in two major crystalline phases: β-Co(O)OH and γ-CoOOH. β-Co(O)OH is typically formed prior to the onset of the OER, while γ-CoOOH, having mixed-valent Co^3+^/Co^4+^ species, is considered the active phase for O–O bond formation. Therefore, the formation of Co^4+^ species is the key for the OER due to its strong affinity for oxygenated intermediates. As a result, for the selective electrooxidation of benzyl alcohol, the formation of Co^4+^ species must be avoided to circumvent the interference of the OER.^9,10^

The Raman spectra of FeCo-HOF-AC and FeCo-LDH-AC detected the E_g_ bending A_1g1_ and A_1g2_ stretching bands for Co^3+^–O. The E_g_ and A_1g1_ bands of FeCo-HOF-AC appeared at higher Raman shift values than those of FeCo-LDH-AC, revealing the higher covalency of the Co–O bond in the former (Fig. 2a).^27,38^ Interestingly, in the case of FeCo-HOF-AC, a third band (A_1g2_) was detected for Co^3+^–O in Co(O)OH. The absence of a separate Fe(O)OH phase is attributed to the preferential leaching of Fe species during the electrochemical activation process. Such Fe leaching has been reported for Fe-containing Co/Ni-based catalysts under strongly alkaline conditions, particularly during the anodic reconstruction of the precatalyst.^38–40^ Therefore, only a relatively small amount of the initially incorporated Fe remains in the reconstructed Co(O)OH framework, where Fe is likely present as a substitutional species rather than forming a separate Fe(O)OH phase.^38,40^

**Fig. 2:**
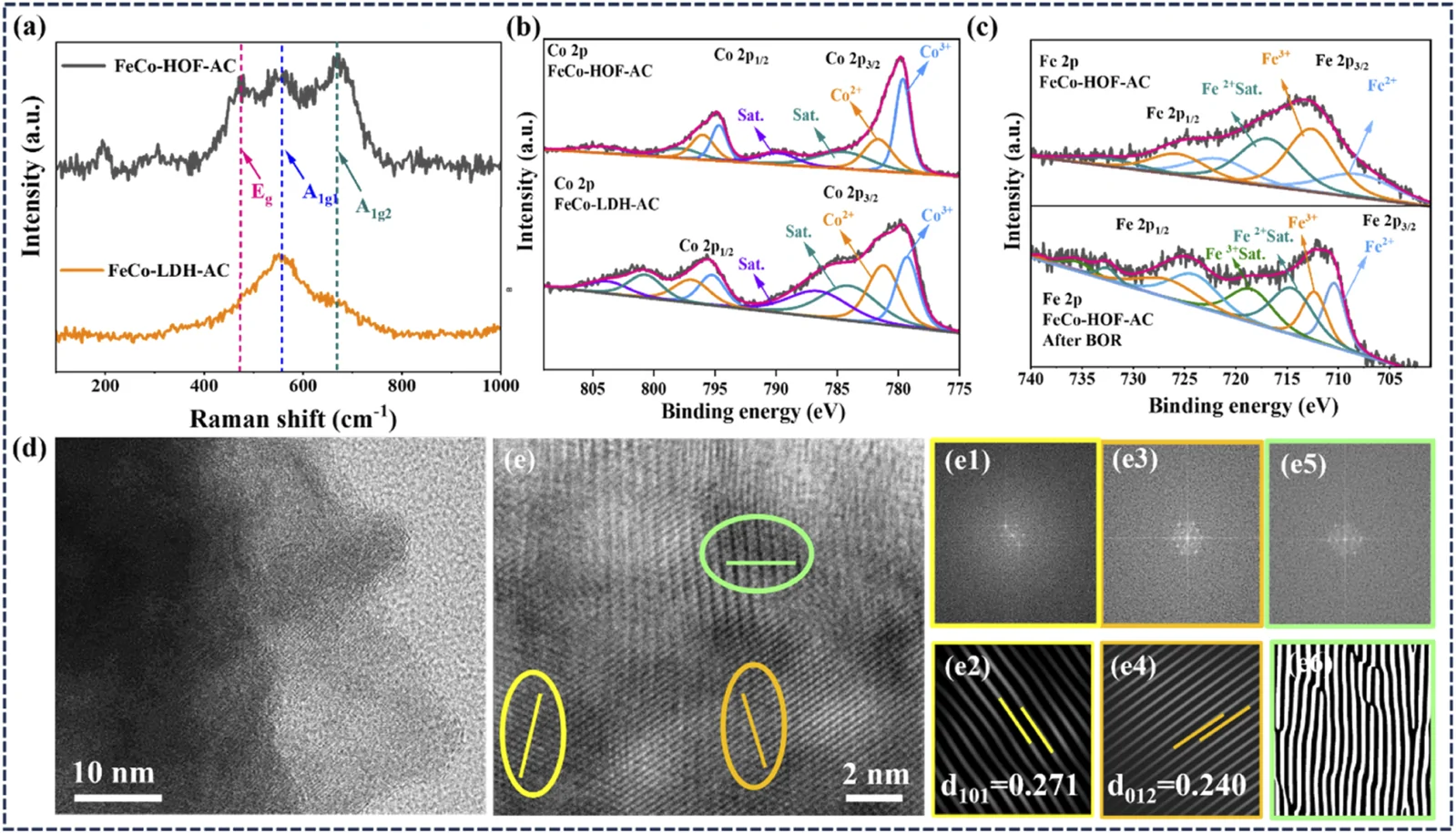
(a) Raman spectra of FeCo-HOF-AC and FeCo-LDH-AC show the bands for Co–O bending (E_g_) and Co–O stretching (A_1g1_ and A_1g2_). (b) Comparison of Co 2p XPS of FeCo-HOF-AC and FeCo-LDH-AC. (c) Comparison of Fe 2p XPS of FeCo-HOF-AC and FeCo-LDH-AC. (d and e) HR-TEM images of FeCo-HOF-AC at different resolutions showed a nanosheet structure with the presence of (012) and (101) planes of β-Co(O)OH. (e1) and (e2) FFT and IFFT of the specified area in figure (e), showing the crystalline structure with the (101) plane of β-Co(O)OH. (e3) and (e4) FFT and IFFT of the specified area in figure (e), showing the crystalline structure with the (012) plane of β-Co(O)OH. (e5) and (e6) FFT and IFFT of the specified area in figure (e), showing the disordered structure.

The XPS study closely matched the conclusions derived from the Raman study. In FeCo-HOF-AC, the Co 2p_3/2_ and 2p_1/2_ peaks were shifted to higher binding energies due to the presence of a higher amount of Co^3+^ species compared to FeCo-LDH-AC (Fig. 2b).^27,41^ FeCo-HOF-AC also exhibited a higher Co^3+^/Co^2+^ peak area ratio (2.6) compared to FeCo-LDH-AC (0.94).

In the active catalysts, the Fe 2p XPS detected the presence of both Fe^2+^ and Fe^3+^ species, while these peaks appeared at a higher binding energy for FeCo-HOF-AC compared to the LDH (Fig. 2c).^42^ Additionally, the Fe^3+^/Fe^2+^ ratio was found to be higher in FeCo-HOF-AC (2.18) than in FeCo-LDH-AC (1.45).

The O 1s XPS of the active catalysts were deconvoluted into peaks for lattice oxygen (O_1_), –OH groups (O_2_), and adsorbed H_2_O molecules (O_3_) (Fig. S15). In FeCo-HOF-AC, the relative amount of lattice oxygen species was found to be higher than that in the precatalyst, and the peak appeared at a higher binding energy compared to that in FeCo-LDH-AC.^42^ This indicates that the HOF structure provides a well-defined Fe/Co coordination environment and facilitates the formation of a highly accessible, porous metal–oxygen framework during electrochemical activation.

CV and XPS analysis conclude that repeated CV cycling progressively transforms Co^2+^ to Co^3+^, increasing the population of Co^3+^-containing active sites in FeCo-HOF-AC. Furthermore, the ratio of the Co^2+^/Co^3+^ redox peak reveals FeCo-HOF with a higher amount of electrochemically accessible active sites, indicating higher availability of Co active centres for successive catalytic oxidation.

The plate-like nanosheet morphology of FeCo-HOF-AC was revealed by the SEM studies (Fig. S16), while the TEM detected thin nanosheets of it (Fig. 2d and S17). HR-TEM images showed lattice spacings of 0.225 nm and 0.271 nm, corresponding to the (101) and (012) crystal planes of β-Co(O)OH (Fig. 2d, e and S17).^26^ Fast Fourier transform (FFT) analyses of different crystalline regions (yellow and orange circles) revealed lattice fringes, confirming the crystalline nature of FeCo-HOF-AC (Fig. 2e). Interestingly, in some regions (green circle), a highly disordered structure was observed, where two adjacent lattice fringes merged into a single lattice. This structural disorder was further validated by the corresponding FFT and inverse FFT (IFFT) analyses (Fig. 2e).^43^

Additionally, the SAED pattern revealed a crystalline structure, with diffraction spots corresponding to the (101) plane of β-Co(O)OH (Fig. S17). The EDX spectroscopy and elemental mapping further confirmed the presence of elements Fe, Co, C, and O within the FeCo(O)OH layers (Fig. S17 and S18).

### Electrocatalytic OER

The initial assessment of the active catalysts for the alkaline OER revealed a far better performance of FeCo-HOF-AC than its LDH analogue, achieving 100 mA cm^−2^ current density at an overpotential of 240 mV (Fig. S19). In comparison, FeCo-LDH-AC required a higher overpotential (295 mV) to reach the same current density. Although the monometallic catalysts Co-LDH-AC and Co–HOF-AC showed poorer OER performance than their bimetallic counterparts, the superior performance of Co–HOF-AC over the LDH-derived catalyst was observed.

Furthermore, Co–HOF exhibits a higher current density of 400 mA cm^−2^ at 1.76 V_RHE_, and the FeCo-HOF achieves this current density at 1.57 V_RHE_, demonstrating the beneficial role of Fe incorporation in improving the OER activity. In addition, we compared the OER activity of FeCo-HOF-AC with that of the hydrothermally synthesised β-Co(O)OH and found that β-Co(O)OH showed comparatively lower OER activity (Fig. S19).

Encouraged by the promising OER performance of FeCo-HOF-AC at room temperature, the electrocatalytic activity was further evaluated at 65 °C. At the elevated temperature, an improvement in the current density at any potential was observed for both the active catalysts compared to room temperature. The superior performance of FeCo-HOF-AC over FeCoLDH-AC was also recorded, while the former reached 100 mA cm^−2^ current density at an overpotential of 210 mV only (Fig. S20).

The increment in the temperature (from 35 °C to 65 °C) also improved the kinetics of the OER for both the active catalysts as revealed by the Tafel analysis (Fig. S21). At both temperatures, slightly faster kinetics for FeCo-HOF-AC over FeCo-LDH-AC were recorded.^44,45^

As a result, the turnover frequency (TOF), an indicator of the catalyst's intrinsic activity, was determined to be significantly higher for FeCo-HOF-AC than for FeCo-LDH-AC. At 65 °C, FeCo-HOF-AC demonstrated a TOF of 461.71 s^−1^, more than 3 times that of FeCo-LDH-AC (144.05 s^−1^).^45^

### Electrocatalytic benzyl alcohol oxidation

The introduction of PhCH_2_OH in the electrolyte solution resulted in a significant improvement in the catalytic current and the shifting of the LSV profiles towards lower anodic potential compared to that observed for the OER. This result clearly showed a lower potential requirement for the BOR than the OER with the same catalyst. FeCo-HOF-AC achieved a current density of 400 mA cm^−2^ at 1.45 V_RHE_, 110 mV lower than the potential required for the OER (1.56 V_RHE_) to reach the same current density (Fig. S20 and S22). The OER activity trend was also followed in the BOR, and FeCo-LDH-AC required a higher potential (1.55 V_RHE_) to reach the same current density (Fig. S22). The higher electrochemical activity of FeCo-HOF-AC can be attributed to the facile transformation of Co^2+^ to Co^3+^ and electron-deficient metal sites (Fe/Co). In addition, we compared the BOR activity of FeCo-HOF-AC with that of hydrothermally synthesised β-Co(O)OH and found that β-Co(O)OH exhibited lower BOR activity (Fig. S22).

At 65 °C, the LSV profile for the BOR reveals the requirement of merely 1.33 V_RHE_ for FeCo-HOF-AC to achieve 400 mA cm^−2^ current density, compared to 1.45 V_RHE_ for FeCo-LDH-AC (Fig. S23). Remarkably, FeCo-HOF-AC delivered a current density of 1.6 A cm^−2^ at only 1.39 V_RHE_, representing one of the lowest operating potentials reported to achieve such a high current density (Fig. 3a, b and Table S3). To the best of our knowledge, the current density achieved at this potential represents the best electrocatalytic PhCH_2_OH oxidation compared to other reported FeCo-based electrocatalysts in the literature (Table S3).

**Fig. 3:**
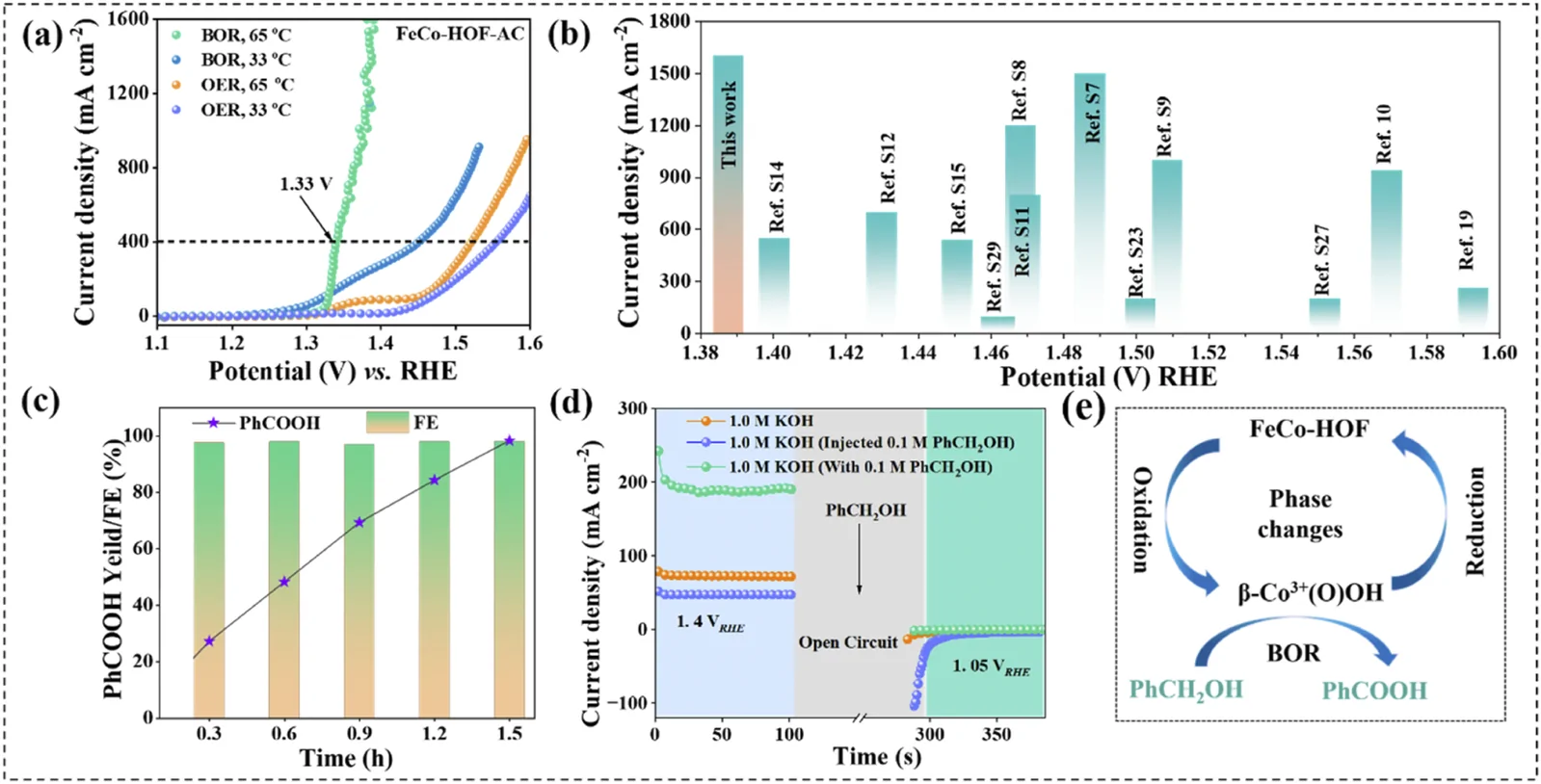
(a) LSV profiles of FeCo-LDH-AC during PhCH_2_OH oxidation at 35 °C and 65 °C. (b) Comparison of the BOR activity of FeCo-HOF-AC with the reported catalysts. (c) Time-dependent conversion and FE plots of FeCo-HOF-AC for PhCH_2_OH oxidation. (d) Multi-step potential curves of FeCo-HOF-AC, recorded in 1.0 M KOH solution in the absence and presence of 0.1 M PhCH_2_OH. (e) Schematic illustration of the BOR pathway over the FeCo-HOF electrocatalyst, highlighting the reversible Co^2+^ to Co^3+^ redox process during PhCH_2_OH oxidation.

When the constant potential (range: 1.32–1.45 V_RHE_) chronoamperometric (CA) method was employed to carry out the PhCH_2_OH oxidation, a wide variation in the FE was observed with increasing potential. For FeCo-HOF-AC, at 1.40 V_RHE_, the PhCOOH yield increased with increasing potential, reaching complete conversion in 1.5 h with 98% FE. However, at higher potentials (1.42 V_RHE_ and 1.45 V_RHE_), the FE decreased sharply (Fig. S24). This indicates that at potentials >1.40 V_RHE_, Co^4+^ sites in FeCo-HOF-AC started participating in the OER, thereby suppressing the BOR activity.

The conversion profile of PhCH_2_OH and the FE of PhCOOH against time were determined under the optimized conditions (1.40 V_RHE_) (Fig. 3c). The formation of benzoic acid by the electrochemical oxidation of PhCH_2_OH was detected by ^1^H and ^13^C NMR studies (Fig. S25).^46^

It is observed that the BOR performance of FeCo-HOF-AC at room temperature is poorer than that at 65 °C (1.40 V_RHE_) (Fig. S25). Under identical conditions, FeCo-HOF-AC achieved only 58% conversion at room temperature, although the FE remained at 97%.

Furthermore, multi-potential step experiments for PhCH_2_OH oxidation and the OER were carried out using FeCo-HOF-AC to elucidate the catalytic mechanism (Fig. 3d).^47,48^ Initially, the potential of the FeCo-HOF-AC anode was set at 1.40 V_RHE_ to generate abundant Co^3+^ species. After holding an open-circuit potential for 90 s, the potential was stepped down to 1.05 V_RHE_. Under OER conditions (without PhCH_2_OH), a pronounced reduction in the current density was observed at 1.05 V_RHE_, indicating the electrochemical reduction of the pre-formed Co^3+^ species, confirming that Co^3+^ is not spontaneously reduced in the absence of a substrate.

In contrast, when PhCH_2_OH was introduced during the open-circuit period, only a weak reduction current appeared at 1.05 V_RHE_, suggesting that the Co^3+^ species were largely consumed through chemical oxidation of PhCH_2_OH. These results demonstrate that the PhCH_2_OH oxidation over FeCo-HOF-AC proceeds *via* a reversible Co^2+^/Co^3+^ redox cycle, where Co^3+^ acts as the active oxidising species (Fig. 3e).

In FeCo-HOF-AC, the ligand-induced stabilization of Co^3+^ and the subsequent inhibition of Co^4+^ species formation improve the BOR selectivity, avoiding any interference from the OER. As a result, we are able to study the BOR activity before the onset potential of the OER (1.43 V _RHE_). At these potentials, the catalytic oxidation primarily involves the participation of Co^3+^ species.

Furthermore, the cycle stability of FeCo-HOF-AC for the BOR was evaluated by CA at room temperature and 65 °C. The FeCo-HOF-AC showed high stability, with the current density remaining stable until the 10th CA cycle (Fig. S26). The FE continued to be at 98% for PhCOOH production throughout 10 consecutive CA cycles.

### Integrated electrochemical hybrid water splitting coupled with PhCH_2_OH oxidation

Based on the above results, a hybrid water electrolyser for PhCH_2_OH oxidation was developed using FeCo-HOF-AC as the anode and NF as the cathode (Fig. S27 and S28). The LSV curve revealed that a current density of 400 mA cm^−2^ for PhCH_2_OH oxidation was achieved at a cell voltage of only 1.71 V. In contrast, the OER required a significantly higher potential of 1.99 V to reach the same current density, demonstrating the superior energy efficiency of the PhCH_2_OH oxidation-assisted water electrolyser. Furthermore, FeCo-HOF-AC attained an exceptionally high current density of 870 mA cm^−2^ at an applied potential of only 1.74 V and achieved 97% FE for PhCOOH (Fig. S28). To the best of our knowledge, no previously reported BOR catalyst has demonstrated such a high current density at a potential below 2.0 V (Table S4).

The above results indicate that the incorporation of chloranilic acid into the FeCo-LDH framework modifies the local electronic structure and surface redox behaviour of the active catalyst. The ligand modification increases the number of catalytically active sites, the amount of Co^3+^ species, and the charge transfer process. Together, these factors increase the OER as well as the BOR activities of FeCo-HOF-AC.

### Structural modification of the active catalyst after benzyl alcohol oxidation

To understand the phase and electronic structural changes after the long-term benzyl alcohol oxidation, PXRD and XPS analyses were performed (Fig. S29–S32).^6,49^ The PXRD pattern showed no change in the phase structure (Fig. S29).

Furthermore, XPS analysis revealed significant changes in the electronic environments of Co, Fe, and O in the active catalyst and after PhCH_2_OH oxidation (Fig. S30–32).^50^ The Co 2p_3/2_ XPS peak of FeCo-HOF-AC-BOR shifted negatively by 0.62 eV compared to that of FeCo-HOF-AC (Fig. S29).^50,51^ Additionally, a decrease in the Co^3+^/Co^2+^ ratio indicated that Co^3+^ species were partially reduced to Co^2+^ during benzyl alcohol oxidation. The Fe 2p XPS also showed the partial reduction of Fe^3+^ to Fe^2+^. After the BOR, the ratio of Fe^3+^ to Fe^2+^ decreased (from 1.45 to 0.88) (Fig. S31).

The decrease in the ratios of Co^3+^/Co^2+^ after the BOR process, compared to the anodically active catalyst, clearly indicates that Co^3+^ ions are actively involved in the PhCH_2_OH oxidation and are reduced to Co^2+^ during the BOR. This change in the oxidation-state distribution provides strong evidence for the participation of the Co^3+^/Co^2+^ redox couple in the BOR catalytic mechanism. This observation further supports the multi-potential step experiments for PhCH_2_OH oxidation (Fig. 3d).

The O 1s spectrum of FeCo-HOF-AC after the BOR exhibited three peaks corresponding to the M–O bonds, surface –OH groups, and adsorbed water molecules, respectively (Fig. S32).^51^ After the BOR, a slight shift of the O 1s spectrum of FeCo-HOF-AC toward a more negative binding energy was observed.

## Conclusions

In conclusion, the modification of FeCo-LDH with the redox-active ligand chloranilic acid results in the formation of FeCo-HOF with a modulated Co^2+^ to Co^3+^ redox couple. The quinonoid structure of chloranilic acid, with electron-withdrawing capability, effectively promotes the oxidation of Co^2+^ to Co^3+^, thereby facilitating the rapid formation of β-Co(O)OH as the active phase. Spectroscopic analyses further confirm significant modulation of the electronic structure of the active centres, providing direct evidence for the origin of the enhanced catalytic performance. As a result, FeCo-HOF-AC achieved a current density of 1.6 A cm^−2^ for benzyl alcohol oxidation at 1.39 V_RHE_ and 65 °C, with 98% faradaic efficiency. Interestingly, in the 2-electrode system, a current density of 400 mA cm^−2^ was achieved at a cell voltage of only 1.71 V for PhCH_2_OH oxidation compared to 1.99 V for the OER, demonstrating the superior energy efficiency of the PhCH_2_OH oxidation-assisted electrolyser.

## Author contributions

T. Ansari was involved in the synthesis, characterisation, data analysis and writing – the original draft. H. Gupta was involved in the synthesis and characterisation. M. Dhillon and A. K. Basu were involved in SEM and TEM characterisation. The planning, design, and supervision of the research, as well as the editing of the manuscript, were carried out by A. Indra.

## Conflicts of interest

There are no conflicts to declare.

## Supplementary Material

SC-OLF-D6SC06869C-s001

## Data Availability

The data that support the findings of this study are available from the corresponding author upon reasonable request. Supplementary information (SI): synthesis and characterization of the catalysts, electrochemical data, and characterization of the products. See DOI: https://doi.org/10.1039/d6sc06869c.
