# Supplementary material for: Ligand-assisted facile Co2+/Co3+ conversion in a metal–hydroxide framework promotes electrochemical benzyl alcohol oxidation at ampere-scale current density
Source: Chem Sci. 2026 Oct 29. Online ahead of print. doi: 10.1039/d6sc06869c (PMC13733532; doi:10.1039/d6sc06869c)
Supplement: Supplementary file 1 [file SC-OLF-D6SC06869C-s001.pdf]

Supplementary Information (SI) for Chemical Science.
This journal is © The Royal Society of Chemistry

Electronic Supplementary Information

Ligand-assisted facile Co^{2+}/Co^{3+} conversion in metal–hydroxide framework promotes electrochemical benzyl alcohol oxidation at Ampere-scale current density

Toufik Ansari,[a] Harshit Gupta,[a] Manshu Dhillon,[b] Aviru Kumar Basu,[b] and Arindam Indra[*,a]

[a]*Department of Chemistry, IIT (BHU), Varanasi, UP-221005, India*
E-mail: arindam.chy@iitbhu.ac.in
[b]*Institute of Nano Science and Technology, (INST), Mohali, India*

Chemicals

All analytical-grade chemicals were used as received without further purification. Nickel foam (NF) was supplied by Axys Technology (West Bengal, India). Ammonium fluoride (NH_4F) and chloranilic acid were purchased from SRL Pvt. Ltd. and Sigma-Aldrich, respectively. Iron(II) sulfate heptahydrate ($FeSO_4{\cdot}7H_2O$), cobalt(II) nitrate hexahydrate ($Co(NO_3)_2{\cdot}6H$₂O), dimethylformamide (DMF), urea (NH_2CONH_2), and potassium hydroxide (KOH) were procured from MERCK Life Sciences Pvt. Ltd. Double-distilled water was used for catalyst synthesis, electrode cleaning, and all electrochemical measurements.

Instrumental

Powder X-ray diffraction (PXRD) patterns of the precatalysts and active catalysts were recorded over a 2θ range of 5° to 70° using a Rigaku D/MAX RINT-2000 diffractometer equipped with Cu Kα radiation (λ = 1.5418 Å).

Fourier-transform infrared (FTIR) spectra were recorded with a Thermo Scientific Nicolet iS5 spectrometer.

Raman spectroscopic (Model: alpha300-RAS) measurements were carried out using a confocal Raman spectrometer.

X-ray photoelectron spectroscopy (XPS) was carried out using a VG/VG ESCA LAB 220i X-ray photoelectron spectrometer to determine the chemical state, oxidation state, and surface electronic structure of the synthesised catalysts. The XPS data were deconvoluted and analysed using Casa software.

The morphology and surface characteristics of the catalysts were examined using a field emission scanning electron microscope (FE-SEM, EVO MA15/18).

Energy-dispersive X-ray (EDX) analysis was performed using the Team Pegasus integrated EDS-EBSD system.

Transmission electron microscopy (TEM) studies were conducted using a Tecnai G2 20 TWIN microscope. For TEM analysis, a small piece of the catalyst film was sonicated in 1 mL of ethanol for 1 hour, and the resulting well-dispersed suspension was drop-cast onto a TEM grid.

Experimental Section

Activation of nickel foam (NF)

The pieces of nickel foams (1 × 2 cm^2) were cleaned with acetone and water. Further, the pieces of nickel foam were treated with 1.0 M HCl for 15 min under ultrasonication conditions to remove the surface impurities. The freshly activated NF was utilised for the synthesis of the catalysts.

Synthesis of nickel-iron layered double hydroxide on nickel foam (FeCo-LDH@NF)

$Co(NO_3)_2{\cdot}6H_2O$ (1.0 mmol), $FeSO_4{\cdot}7H_2O$ (1.0 mmol), NH_4F (4.0 mmol), and NH_2CONH_2 (10.0 mmol) were dissolved in 12 mL of water and stirred for 30 min. The nickel foam pieces were placed vertically in the solution into a Teflon-lined autoclave and sealed. The autoclave was heated at 120 °C for 5 h. After cooling

to room temperature, the FeCo-LDH@NF films were washed with water, then with ethanol, and dried in an air oven at 50 °C for 12 h.

The $Co(OH)_2$@NF films were prepared using a method similar to that for FeCo-LDH@NF, using only $Co(NO_3)_2 \cdot 6H_2O$ (2.0 mmol) as the metal ion source.

Synthesis of FeCo-HOF@NF

For the synthesis of FeCo-HOF@NF, 32 mg of chloranilic acid was dissolved in 11 mL of mixed solution of DMF and water (10:1). The two pieces of FeCo-LDH@NF were immersed vertically into the solution in a Teflon-lined autoclave. The autoclave was sealed and placed in a preheated air oven at 100 °C for 5 h. After the normal cooling, the synthesised FeCo-HOF@NF films were washed with DMF and ethanol several times. The films were further dried in an air oven at 50 °C overnight.

For comparison purposes, Co-HOF@NF was also synthesised using a similar method by immersing $Co(OH)_2$@NF into the solution of chloranilic acid.

Synthesis of β-$Co(OH)_2$

β-$Co(OH)_2$ was prepared by dissolving 3.1249 g of $Co(NO_3)_2 \cdot 6H_2O$ in 20 mL of water and stirred for 15 minutes. Then, 10 mL of a 2 M NaOH solution was added, and the mixture was stirred for 15 minutes. The solution was transferred to a clean Teflon-lined stainless-steel autoclave. The autoclave was sealed and placed in a preheated air oven at 120 °C for 2 h. After reaching room temperature, the precipitate was washed with water and dried at 60 °C for 24 hours.

Synthesis of β-Co(O)OH[1]

For the synthesis of β-Co(O)OH, the collected $Co(OH)_2$ was redispersed in 15 mL of 8 M NaOH under magnetic stirring at 45 °C, and then 6 mL of 30% H_2O_2 solution was added dropwise. After that, the reaction is kept for 24 h at 45 °C. The Co(O)OH sample is collected by centrifugation at 4000 rpm.

Table S1. Details of synthesised catalysts

Precatalyst	Precursor(s)	Chloranilic acid
FeCo-LDH	$FeSO_4.7H_2O$ (1 mmol)<br>$Co(NO_3).6H_2O$ (1 mmol)	---
Co-LDH	$Co(NO_3).6H_2O$ (2 mmol)	---
FeCo-HOF	FeCo-LDH@NF	32 mg
Co-HOF	Co-LDH@NF	32 mg

Electrochemical measurements

The electrochemical measurements were carried out in a single-compartment three-electrode electrochemical cell in 1.0 M aqueous KOH solution (otherwise mentioned in the caption of the figure). The HOF and LDH films on nickel foam were employed as the working electrode, and Pt wire as the counter electrode. The Hg/HgO electrode was used as the reference electrode in 1.0 M aqueous KOH solution (pH 13.6). Linear sweep voltammetry (LSV) was performed and presented with 70% i*R* compensation. All the potentials were presented against reversible hydrogen electrode (RHE) using the formula:

$$E_{RHE} = E_{Hg/HgO} + 0.098 + \frac{2.303 \times RT\ (pH)}{F}$$

Where F represents the Faraday constant (96,485 C mol^{-1}), R represents the universal gas constant (8.314 J. $mol^{-1} \cdot K^{-1}$), and T denotes the temperature. Corresponding temperature correction was carried out when the reactions were performed at varying temperature.

Electrochemical impedance spectroscopic (EIS) measurements were recorded in the frequency range from 0.001 to 100,000 Hz and amplitude of 10 mV.

Tafel plots were determined by potentiostatic measurements at the potentials where current density reached up to 50 mA cm^{-2}. The Tafel slope was calculated by using the Tafel equation:

$$\square = b \log j + a$$

Where, η: overpotential (V), j: current density (A cm^{-2}), and b: Tafel slope (V dec^{-1}).

For C_{dl} measurements, CV was carried out at a potential range (0.8 to 0.9 V_{RHE}), where no faradaic process occurred.

Analysis of product(s) by NMR

After complete passage of charge, the oxidised product was isolated by neutralisation and hot-water crystallisation. The organic product formation after the electro-oxidation reaction of $PhCH_2OH$ was analysed by ^{1}H and ^{13}C NMR spectroscopy. The NMR sample was prepared by taking 5 mg of the reaction product and 500 μL $CDCl_3$. Both ^{1}H and ^{13}C NMR spectra were analysed using MestReNova software.

Calculation of Faradaic efficiency

The FE were calculated using the equation below:

$$FE\ (\%) = \frac{\text{Mole of product produced}}{\text{Total charge passed}/(n \times 96485)} \times 100$$

Where, F represents the Faraday constant (96,485 C mol^{-1}), and n denotes the number of electrons involved in the oxidation reaction (4 e^-). After 1.5 h of electrolysis, the potentiostat recorded a total charge of 769.64 C during BOR. Based on the experimental values, a final FE of 98 % was obtained after 1.5 h of reaction.

Figures

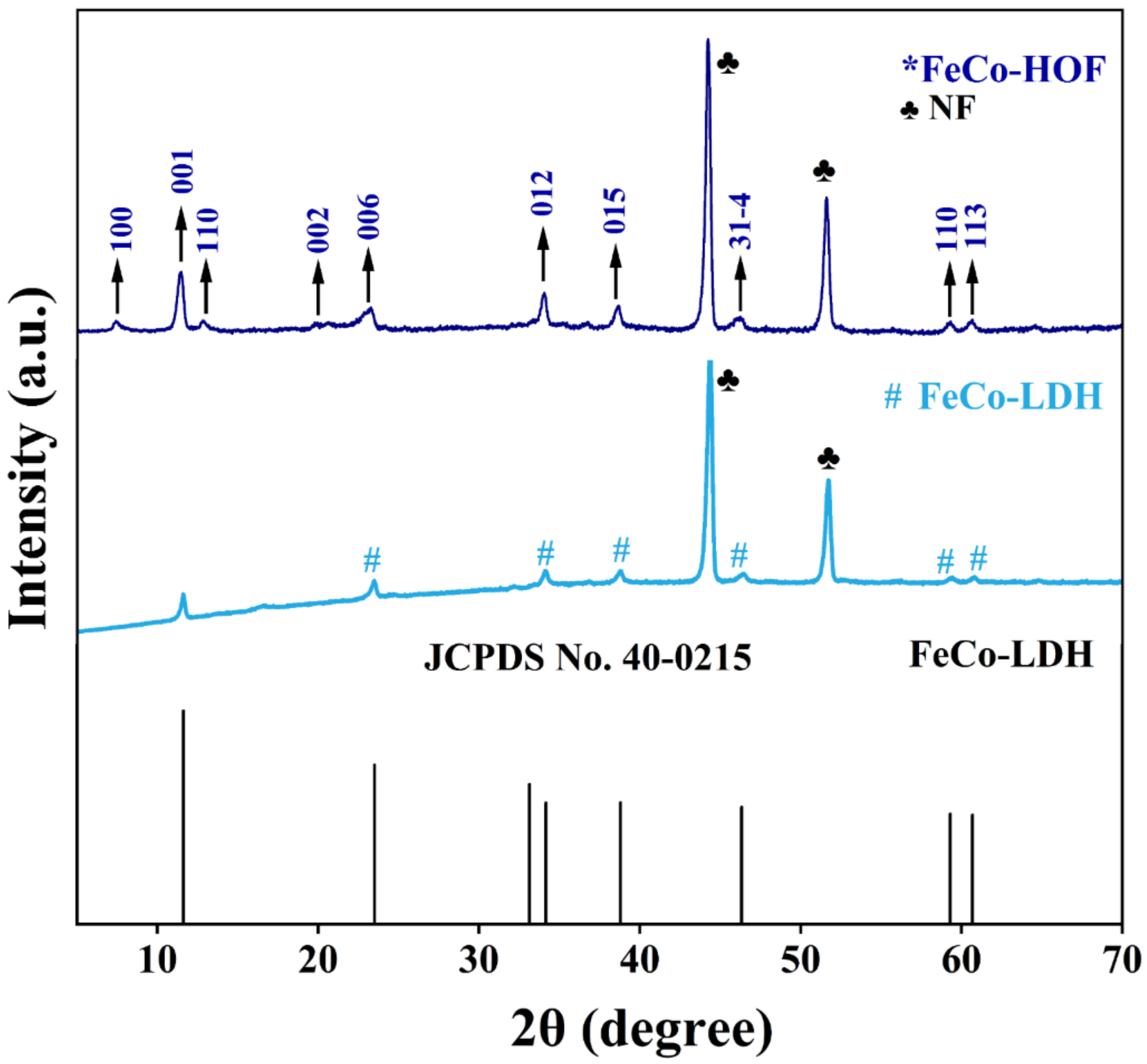

Figure S1. PXRD patterns of FeCo-HOF@NF and FeCo-LDH@NF. All diffraction peaks of FeCo-LDH are well matched with the standard JCPDS card no. 40-0215, while the diffraction pattern of FeCo-HOF is matched with that of previously reported NiFe-HOF.[2-3]

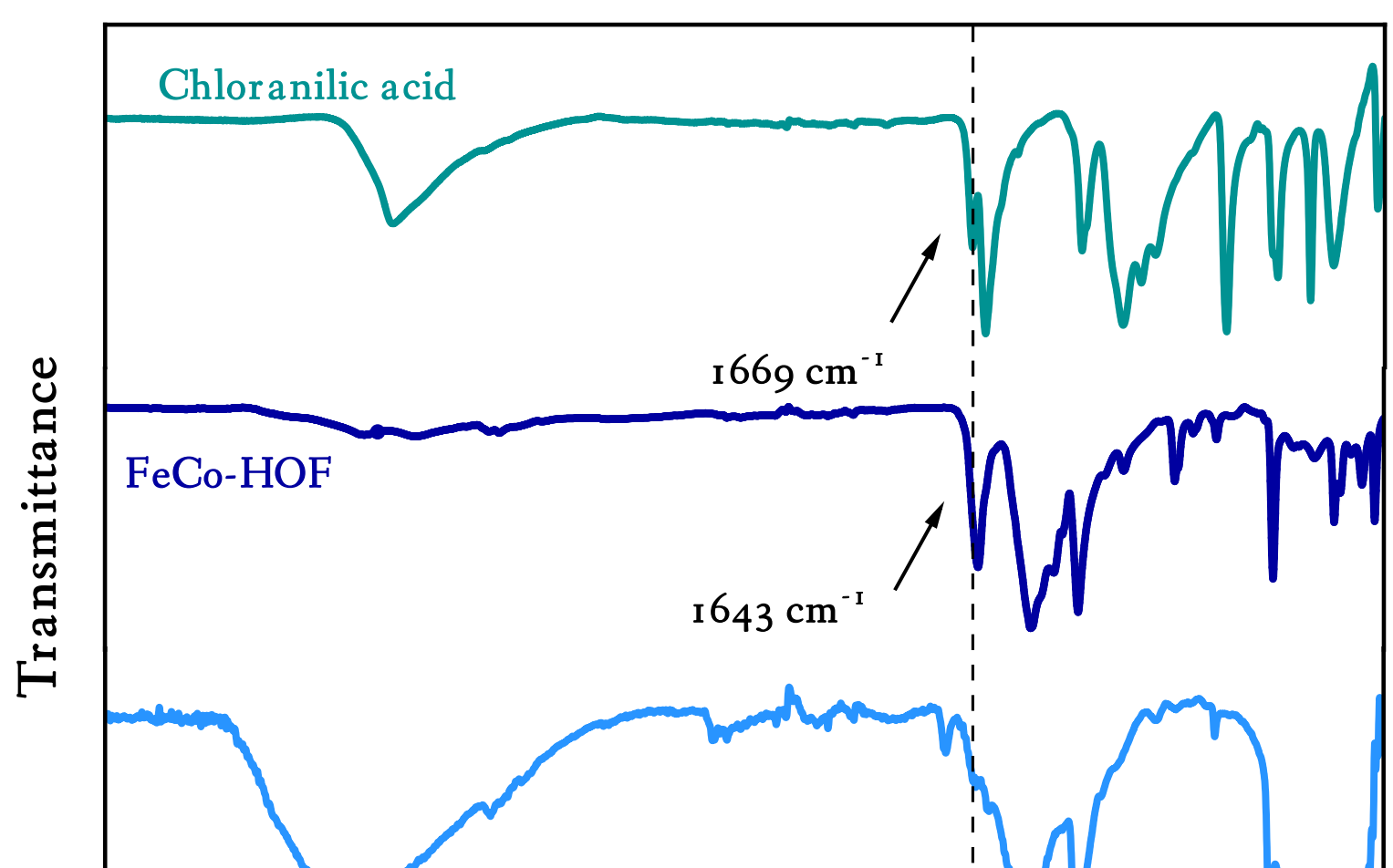

Figure S2. The attenuated total reflection Fourier-transformed infrared (ATR-FTIR) spectrum of FeCo-HOF@NF, FeCo-LDH@NF, and free chloranilic acid. The peaks at 1643 cm^{-1} and 1669 cm^{-1} were assigned to the stretching vibrations of the C=O group in FeCo-HOF@NF and free chloranilic acid, respectively.[2,4] The red shift of the C=O vibrations, relative to the peak in free chloranilic acid, was observed due to the delocalisation of the π-electrons in the bidentate chloranilate anion coordinated metal centres.

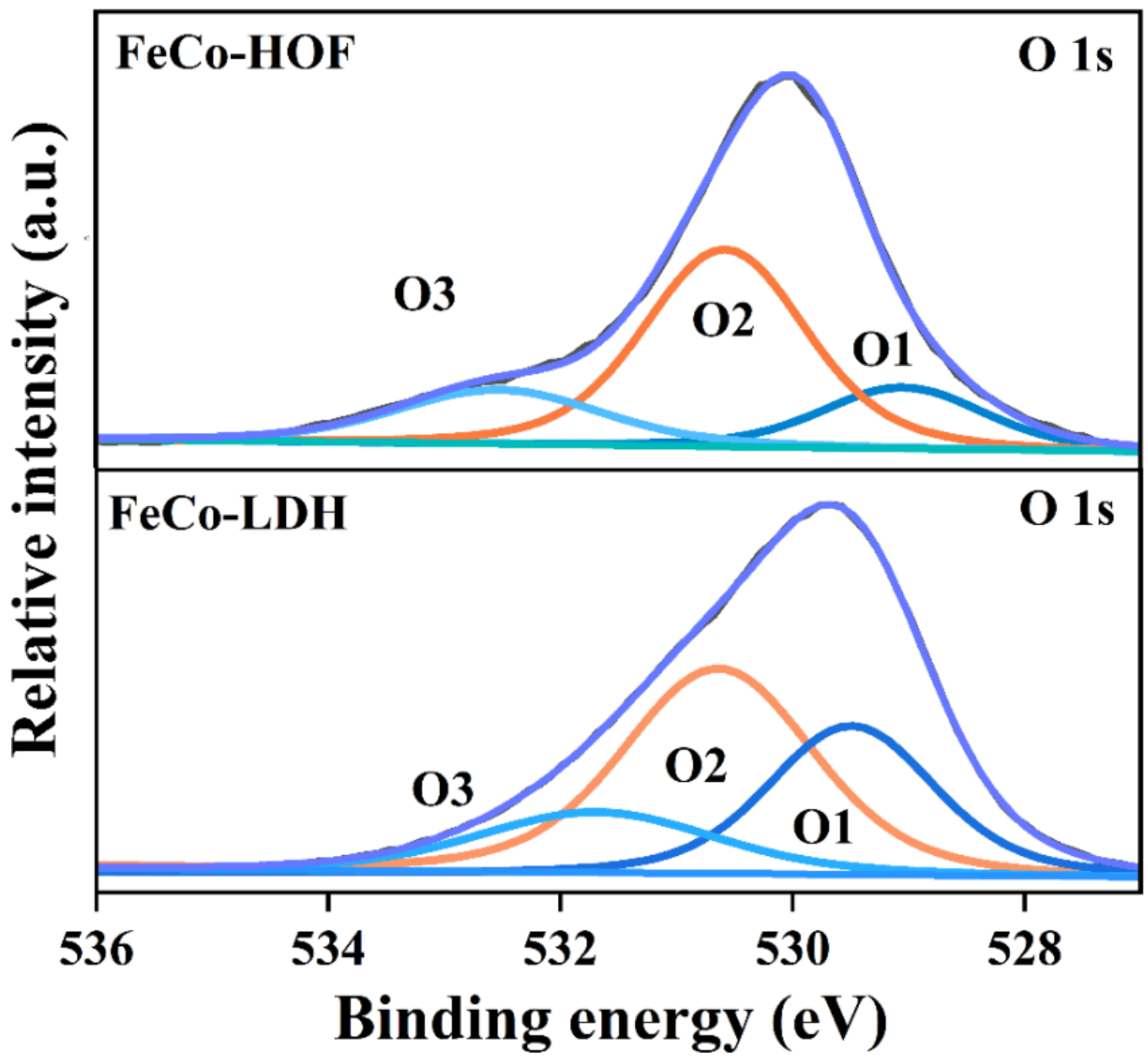

Figure S3. O 1s XPS of FeCo-LDH@NF and FeCo-HOF were deconvoluted into three different oxygen species, O1: lattice oxygen, O2: –OH group, and O3: absorbed water molecules.[4-5]

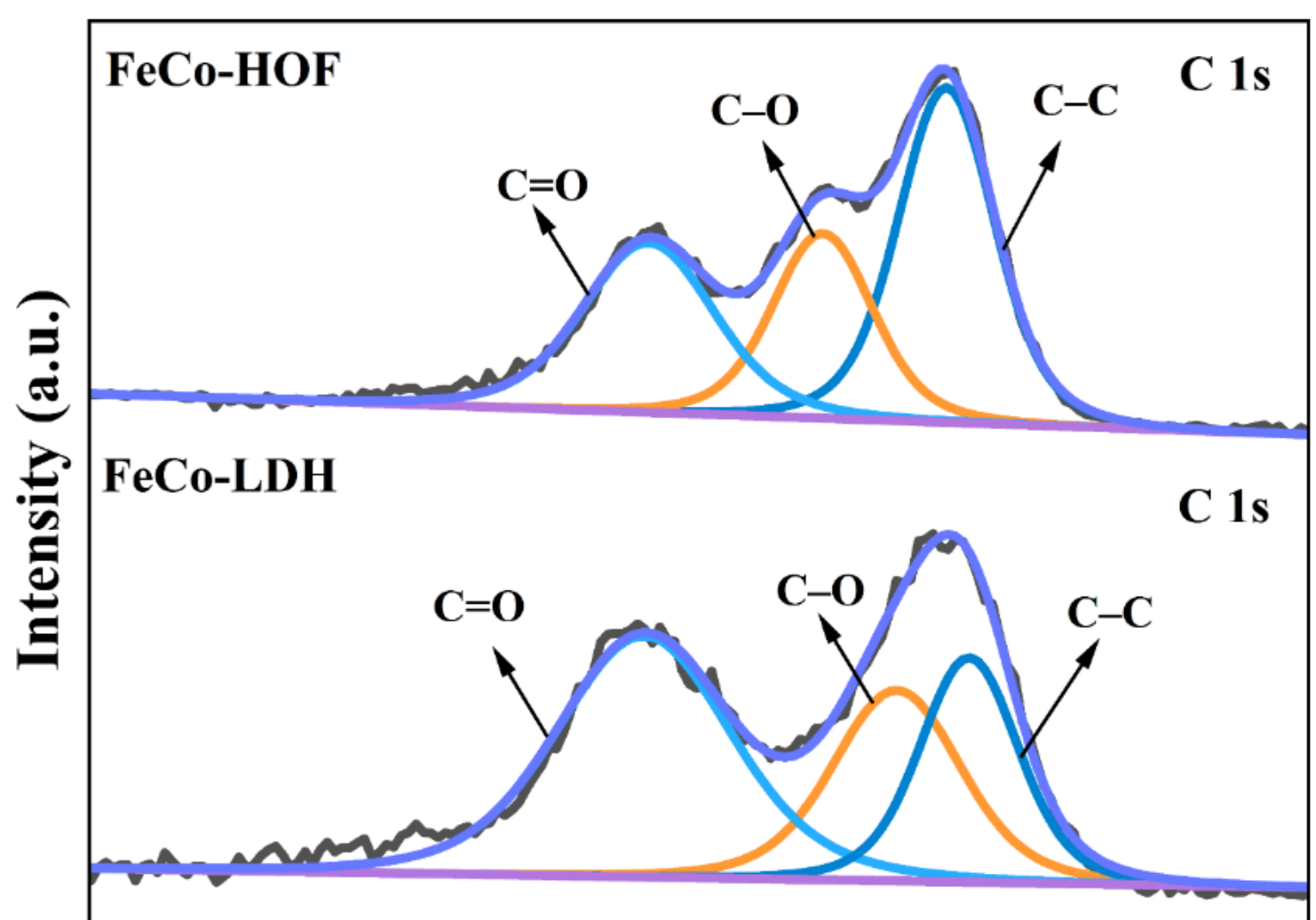

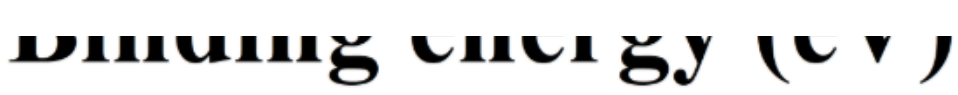

Figure S4. C 1s XPS of FeCo-HOF@NF and FeCo-LDH@NF. FeCo-HOF@NF showing three peaks at 284.91 eV (C-C), 285.22eV (C-O), and 288.90 eV (C=O). Similarly, FeCo-LDH@NF also shows three peaks corresponding to (C-C), (C-O), and (C=O).[4-5]

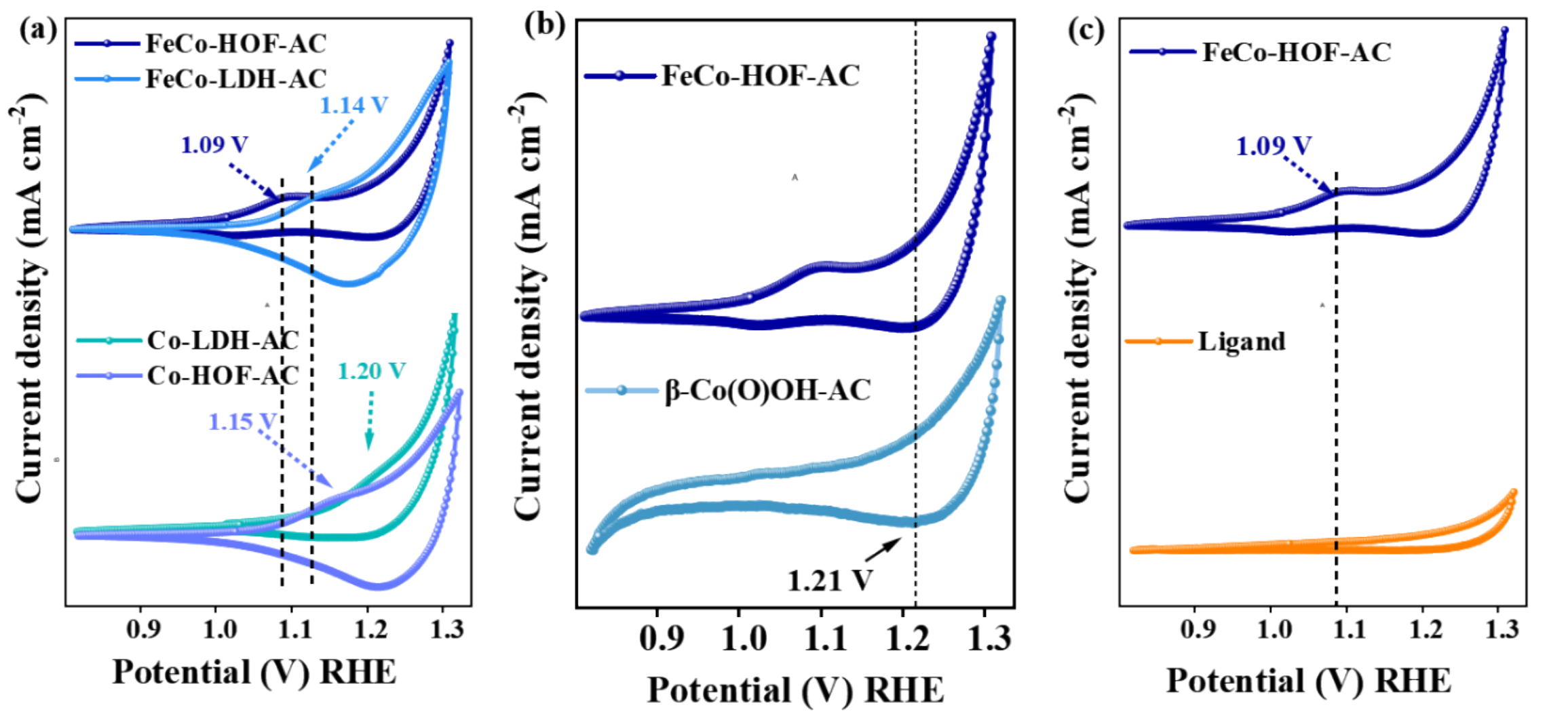

Figure S5: (a) The CV profile of the precatalysts showed the oxidation peaks in the low-potential window (1.00–1.30 VRHE), indicating the oxidation of Co^{2+}/Co^{3+}. (b) Comparison of the CV profiles of FeCo-HOF and the β-C(O)OH. (b) Comparison of the CV profiles of FeCo-HOF and the chloranilic acid ligand.

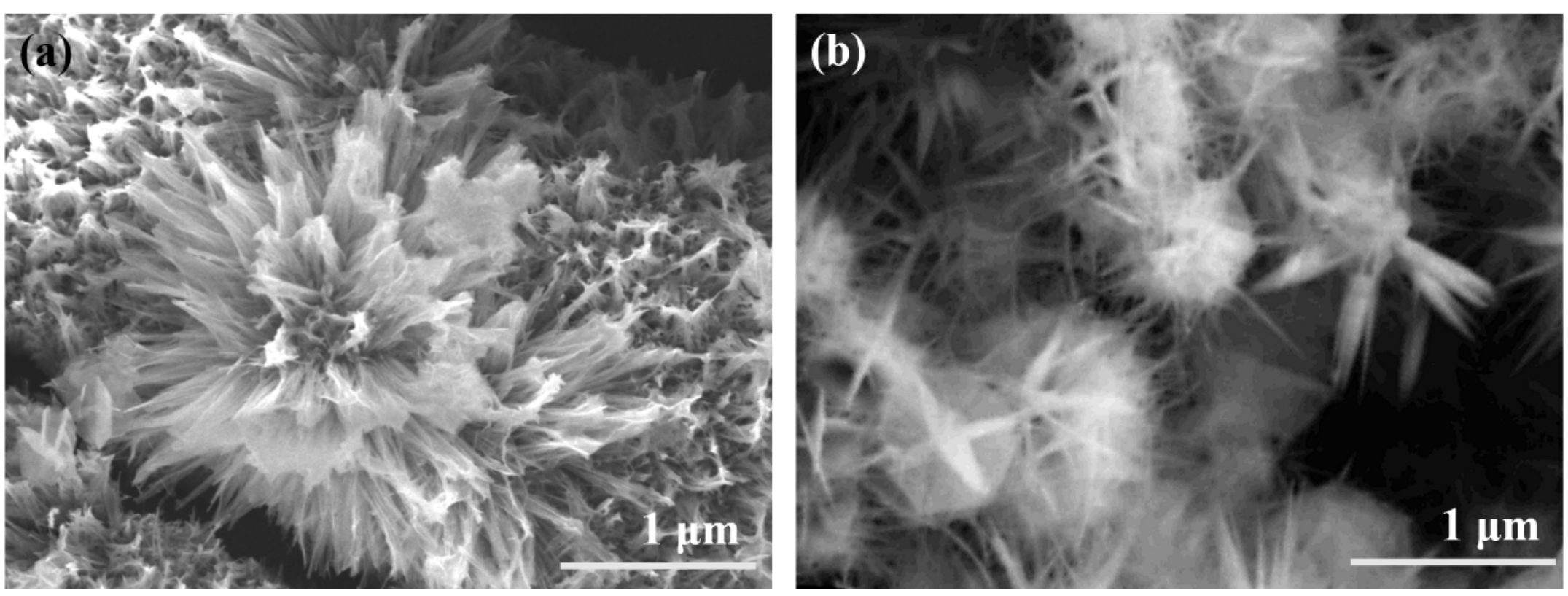

Figure S6. FESEM image of (a) FeCo-LDH and (b) FeCo-HOF showing the flower-like morphology.

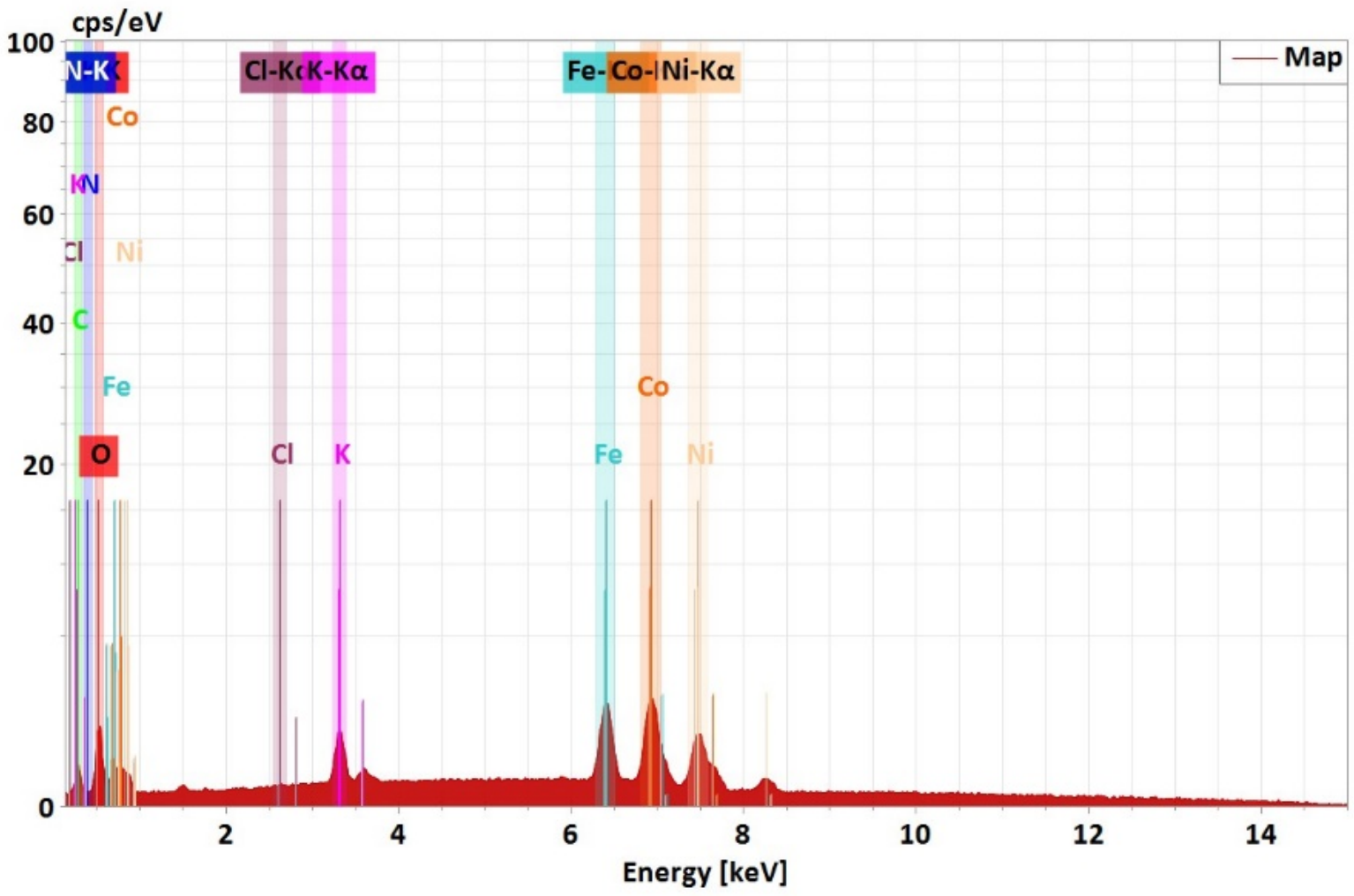

Figure S7. EDX spectrum of FeCo-HOF, indicating the presence of the elements Co, Fe, C, Cl, and O. The Ni peak comes from the Ni foam support.

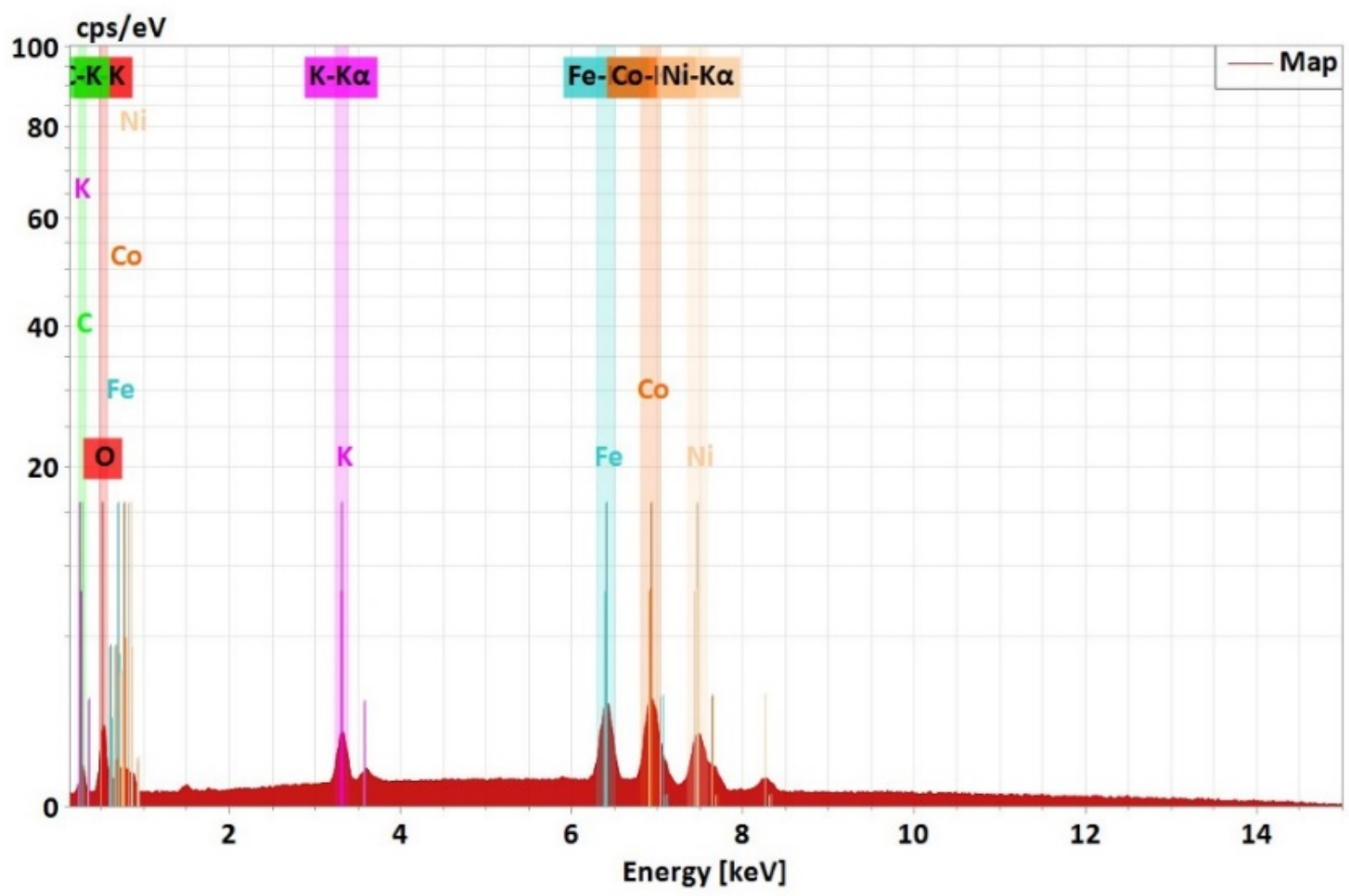

Figure S8. EDX spectrum of FeCo-LDH indicating the presence of the elements Co, Fe, C, and O. The Ni peak comes from the Ni foam support.

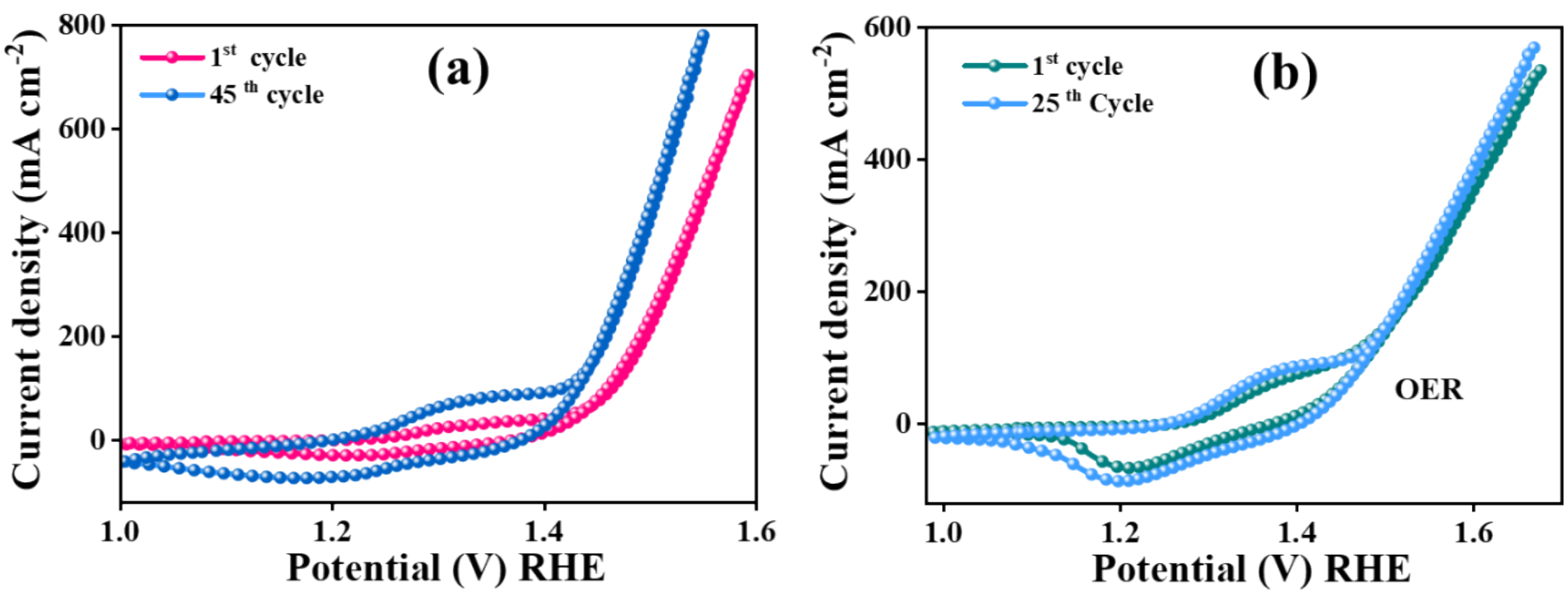

Figure S9. CV profiles for the activation of (a) FeCo-HOF and (b) FeCo-LDH.

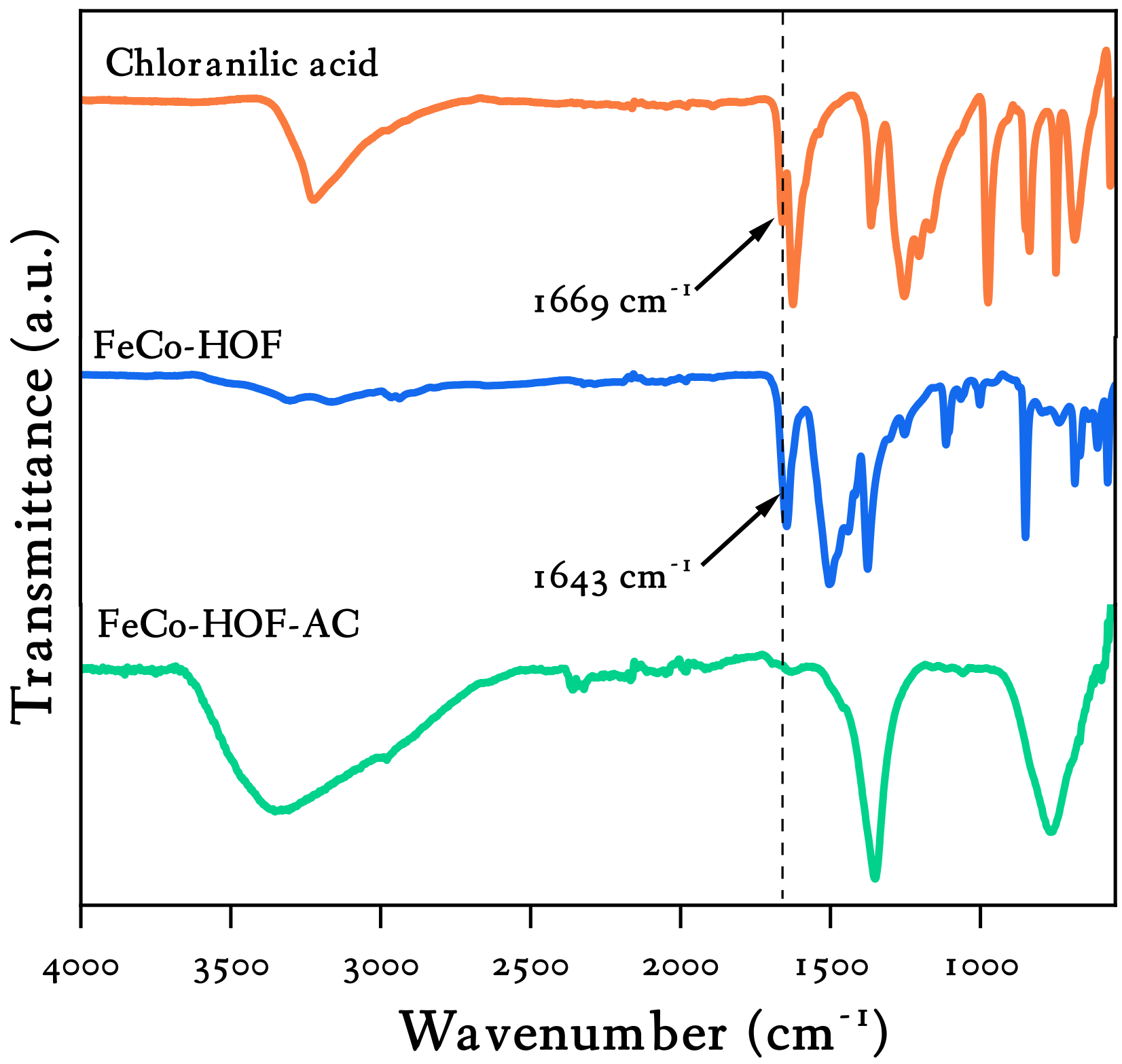

Figure S10. The attenuated total reflection Fourier-transformed infrared (ATR-FTIR) spectrum of FeCo-HOF, chloranilic acid active catalyst FeCo-HOF-AC, showing that the peaks for C=O and C-O vibrations had disappeared after electrochemical activation.[1-3]

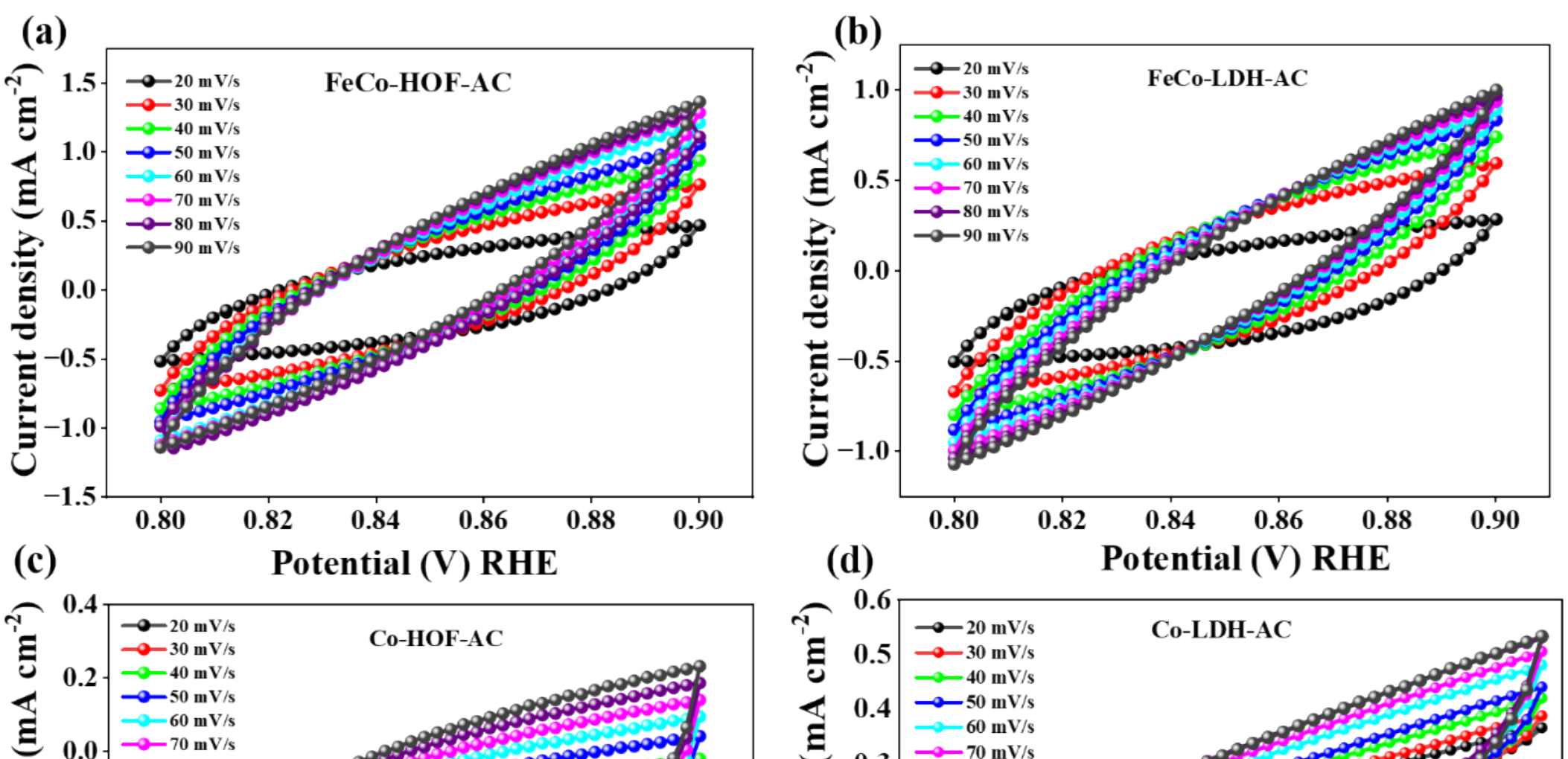

Figure S11. CV plots for (a) FeCo-HOF-AC; (b) FeCo-LDH-AC, (c) Co-LDH-AC and (d) Co-HOF-AC in the non-faradaic region; (e) electrochemical double-layer capacitance C_{dl} value for FeCo-HOF-AC, FeCo-LDH-AC, Co-LDH-AC, and Co-HOF-AC.

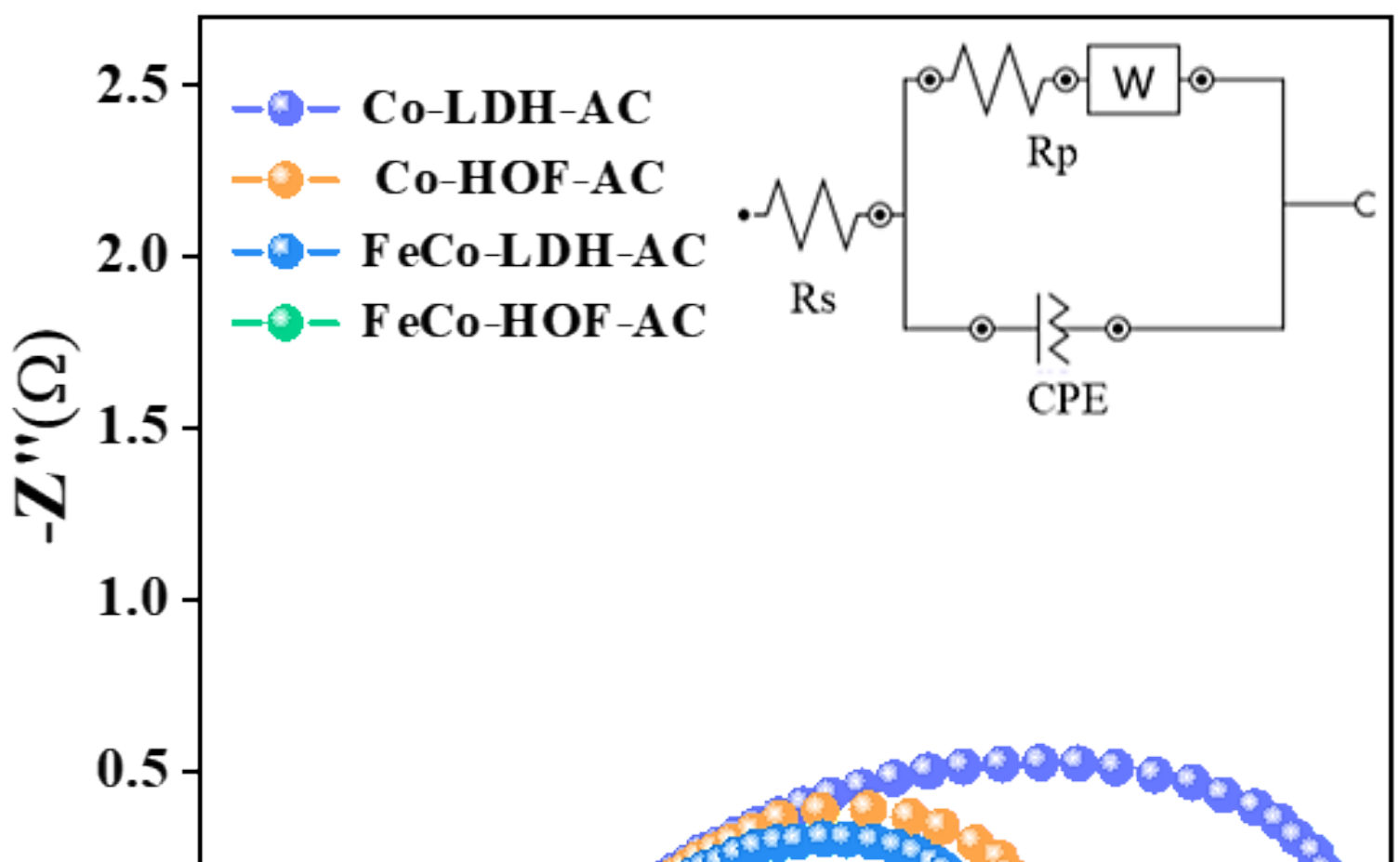

Figure S12. Nyquist plots for the FeCo-HOF-AC, FeCo-LDH-AC, Co-HOF-AC and Co-LDH-AC, showing the lowest charge transfer resistance for FeCo-HOF-AC. The frequency range of EIS measurement was 0.01 to 10^6 Hz at 1.5 V_{RHE}.

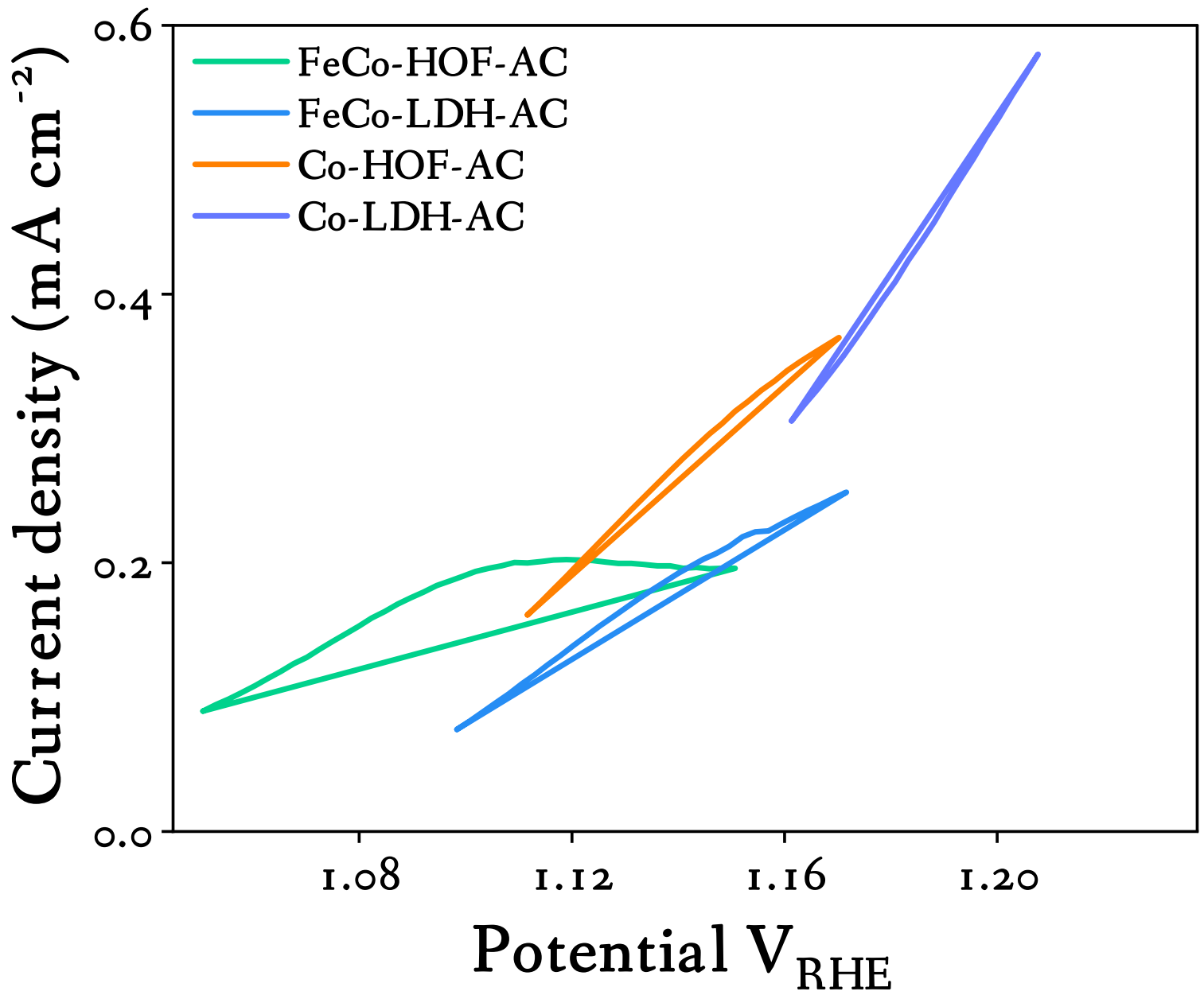

Figure S13. Oxidation peak area of FeCo-HOF-AC, FeCo-LDH-AC, Co-HOF-AC, and Co-LDH-AC utilized for integration to determine the number of surface-active sites. [1,5]

Determination of surface-active sites using area integration of the oxidation peak in Figure S12.[1]

FeCo-HOF-AC

Calculated area associated with the reduction peak = 0.0027×10^{-3} V A

Hence the associated charge is = 0.0027×10^{-3} V A / 0.005 V s^{-1}

= 0.541×10^{-3} A s = 0.541×10^{-3} C

Now, the number of electron transferred is = 0.541×10^{-3} C / 1.602×10^{-19} C

$= 3.38 \times 10^{15}$

Since the oxidation of Co^{2+} to Co^{3+} is a single-electron transfer reaction, the number of electrons calculated above is the same as the number of surface-active sites.

Hence, the surface-active Co^{3+} sites participated in OER is $= 3.38 \times 10^{15}$

FeCo-LDH-AC

Calculated area associated with the reduction peak $= 0.000632\times 10^{-3}$ V A

Hence the associated charge is $= 0.000632\times 10^{-3}$ V A / 0.005 V s^{-1}

$= 0.126 \times 10^{-3}$ A s

$= 0.126 \times 10^{-3}$ C

Now, the number of electron transferred is $= 0.126 \times 10^{-3}$ C / 1.602×10^{-19} C

$= 0.78 \times 10^{15}$

The surface-active Co^{3+} sites participated in OER is $= 0.78 \times 10^{15}$

Co-HOF-AC

Calculated area associated with the reduction peak $= 0.000491\times 10^{-3}$ V A

Hence the associated charge is $= 0.000491\times 10^{-3}$ V A / 0.005 V s^{-1}

$= 0.0982\times 10^{-3}$ A s

$= 0.0982\times 10^{-3}$ C

Now, the number of electron transferred is $= 0.0982\times 10^{-3}$ C / 1.602×10^{-19} C

$= 0.61 \times 10^{15}$

The surface-active Co^{3+} sites participated in OER is $= 0.61 \times 10^{15}$

Co-LDH-AC

Calculated area associated with the reduction peak $= 0.000630\times 10^{-3}$ V A

Hence the associated charge is $= 0.000310\times 10^{-3}$ V A / 0.005 V s^{-1}

$= 0.062 \times 10^{-3}$ A s

$= 0.062 \times 10^{-3}$ C

Now, the number of electron transferred is $= 0.062 \times 10^{-3}$ C / 1.602×10^{-19} C

$= 0.38 \times 10^{15}$

The surface-active Co^{3+} sites participated in OER is $= 0.38 \times 10^{1}$

Table S2. Comparison of the electrochemical properties of the active catalysts.

**Catalysts**	**R_{ct} value (Ω)**	**Number of surface active Co^{3+} species**	**C_{dl} value (mF dec^{-1})**
FeCo-HOF-AC	0.54	3.38×10^{15}	10.10
FeCo-LDH-AC	0.94	0.78×10^{15}	9.21
Co-HOF-AC	1.09	0.61×10^{15}	4.71

Co-LDH-AC	1.82	0.38×10^{15}	2.37

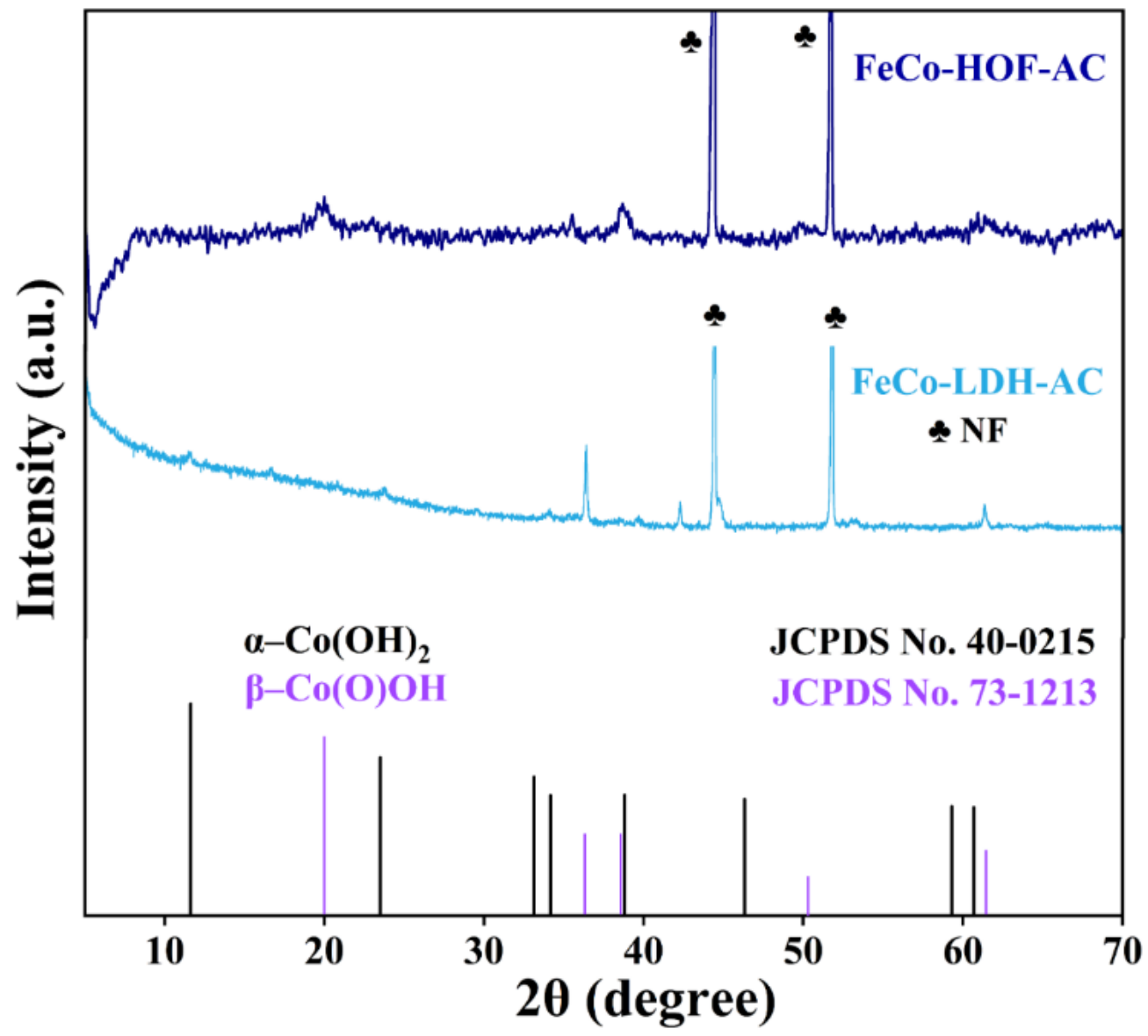

Figure S14. PXRD patterns of the active catalysts FeCo-HOF-AC and FeCo-LDH-AC. [6]

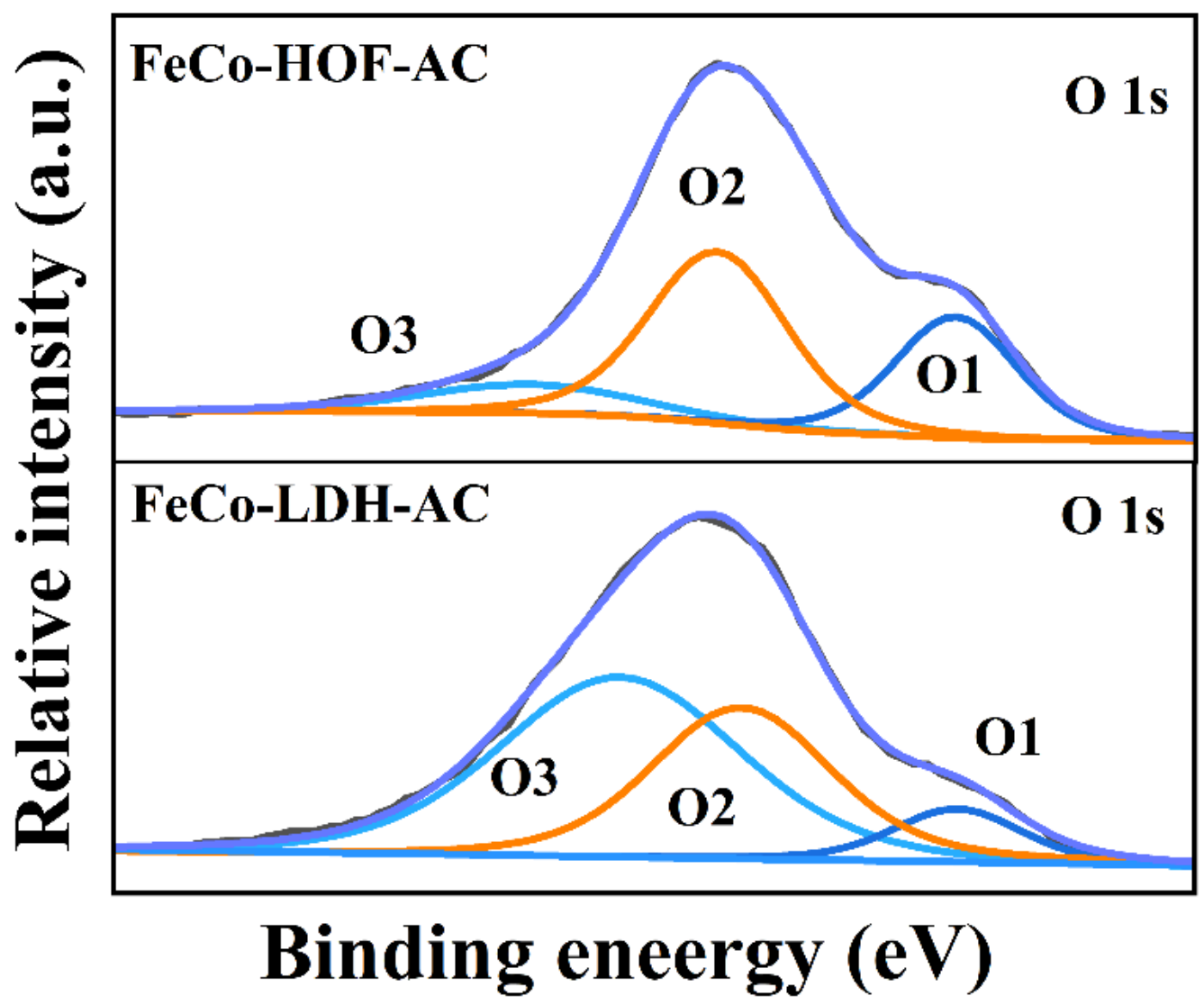

Figure S15. O 1s XPS of FeCo-HOF-AC and FeCo-LDH-AC were deconvoluted into O1: lattice oxygen, O2: –OH group, O3: absorbed water molecules.[5-6]

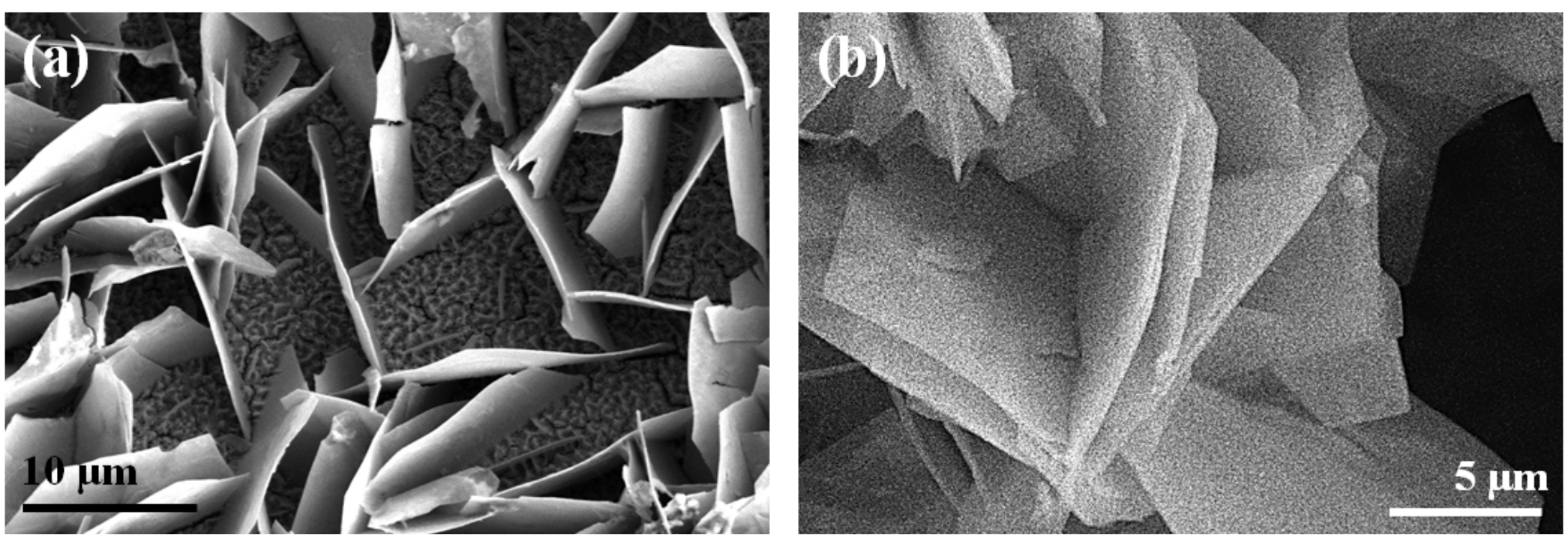

Figure S16. (a-b) FESEM images of the active catalyst FeCo-HOF-AC showing the nanoplate morphology.

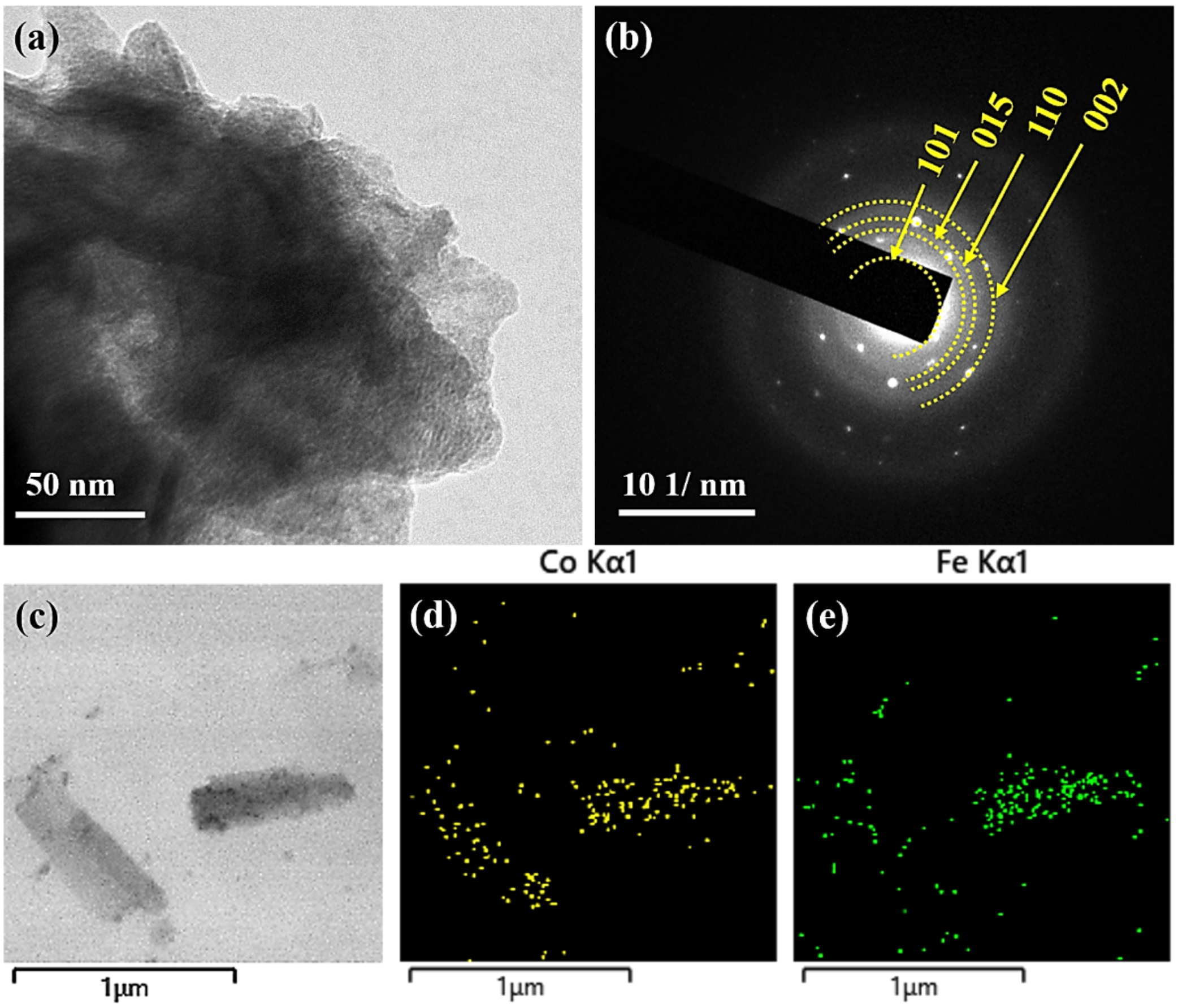

Figure S17. (a) TEM image of the active catalyst FeCo-HOF-AC, showing the nanosheet morphology. (b) The selected area electron diffraction (SAED) pattern of FeCo-HOF-AC was indexed for the (101), (015), (110), and (002) planes of β-Co(O)OH. (c-e) Elemental mapping showing the homogeneous distribution of Co and Fe.

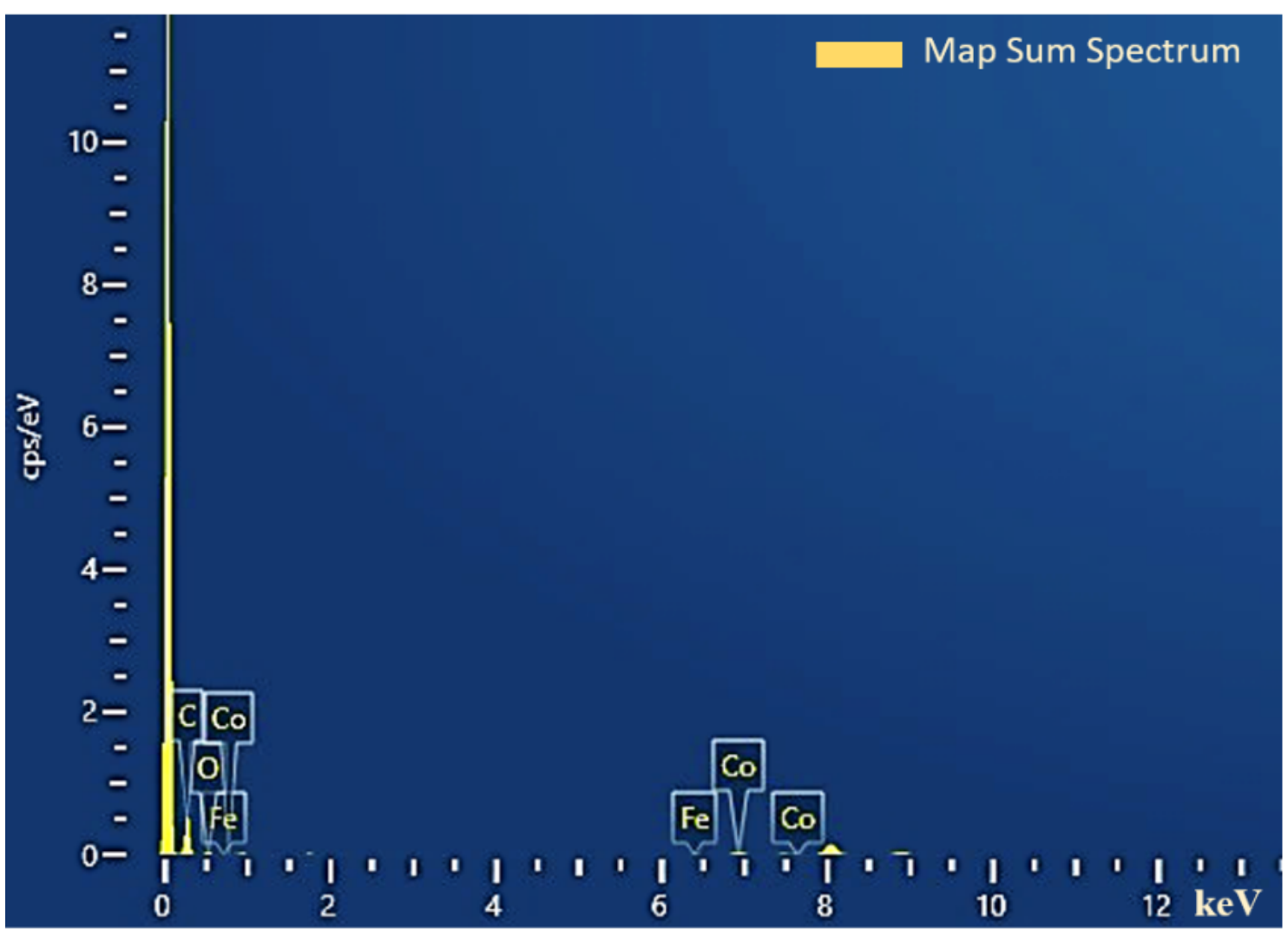

Figure S18. EDX spectrum of FeCo-HOF-AC, showing the elements Co, Fe, C, and O.

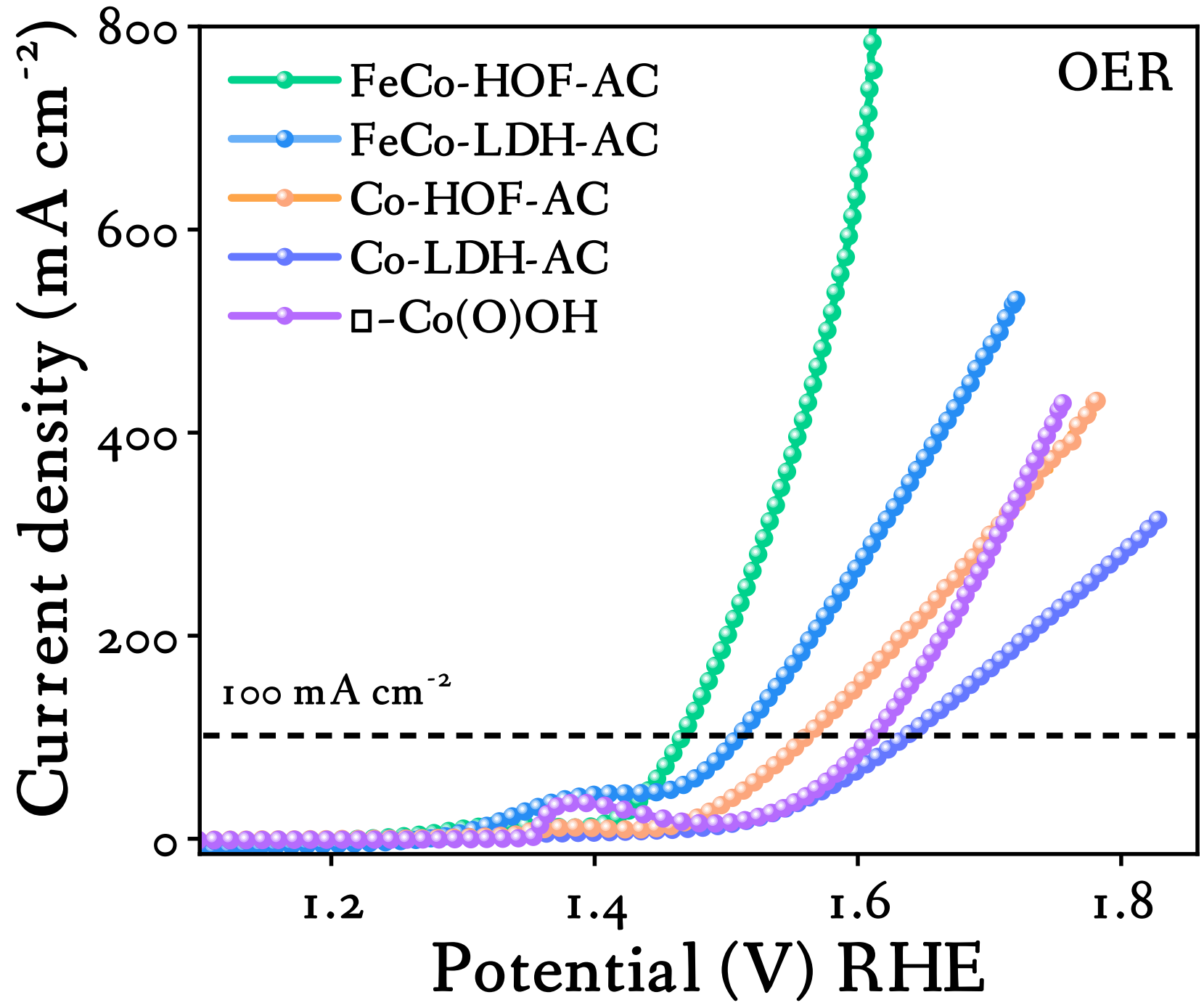

Figure S19. LSV profiles for OER with FeCo-HOF-AC, FeCo-LDH-AC, Co-HOF-AC and Co-LDH-AC at room temperature.

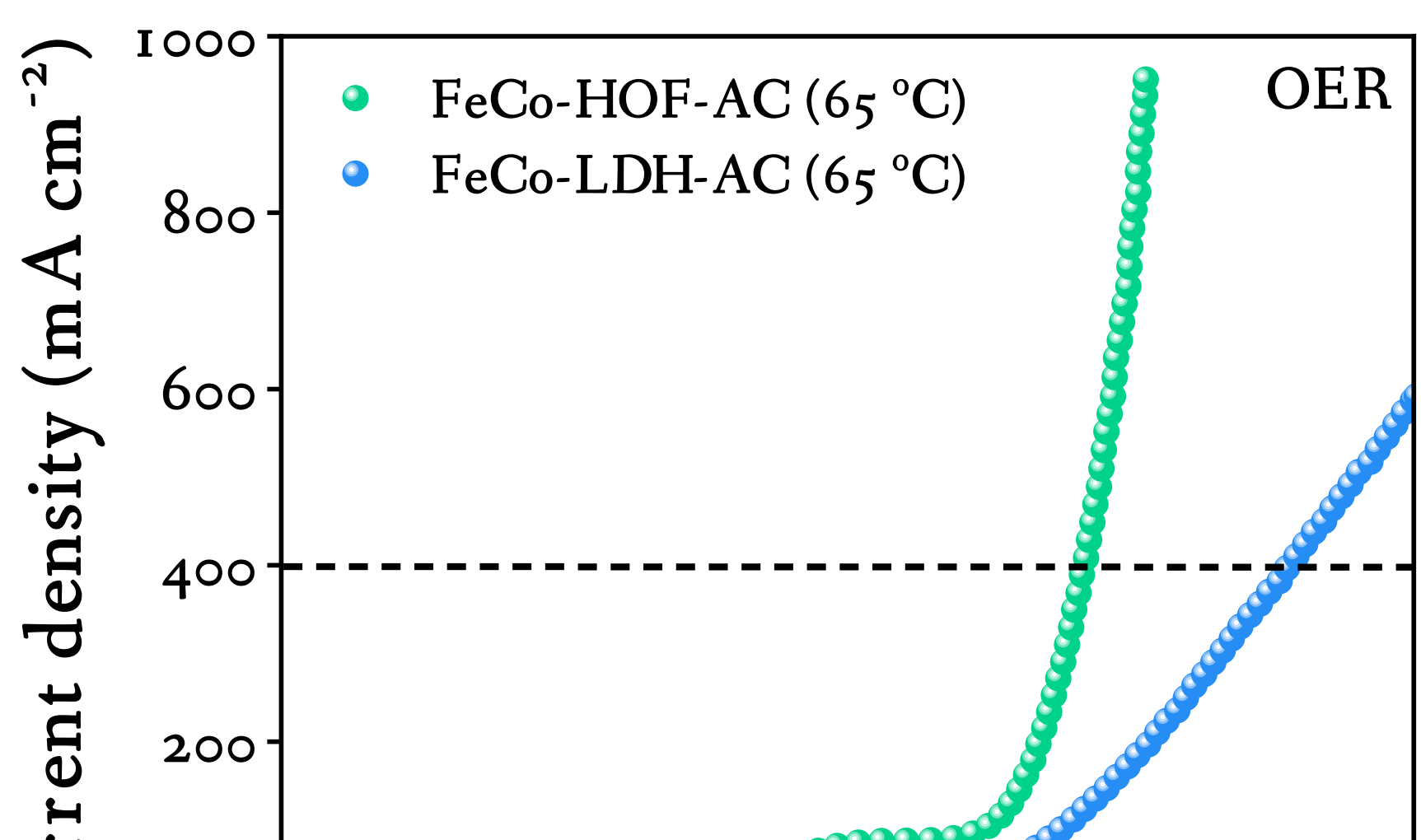

Figure S20. LSV profiles for OER with FeCo-HOF-AC and FeCo-LDH at 65 °C.

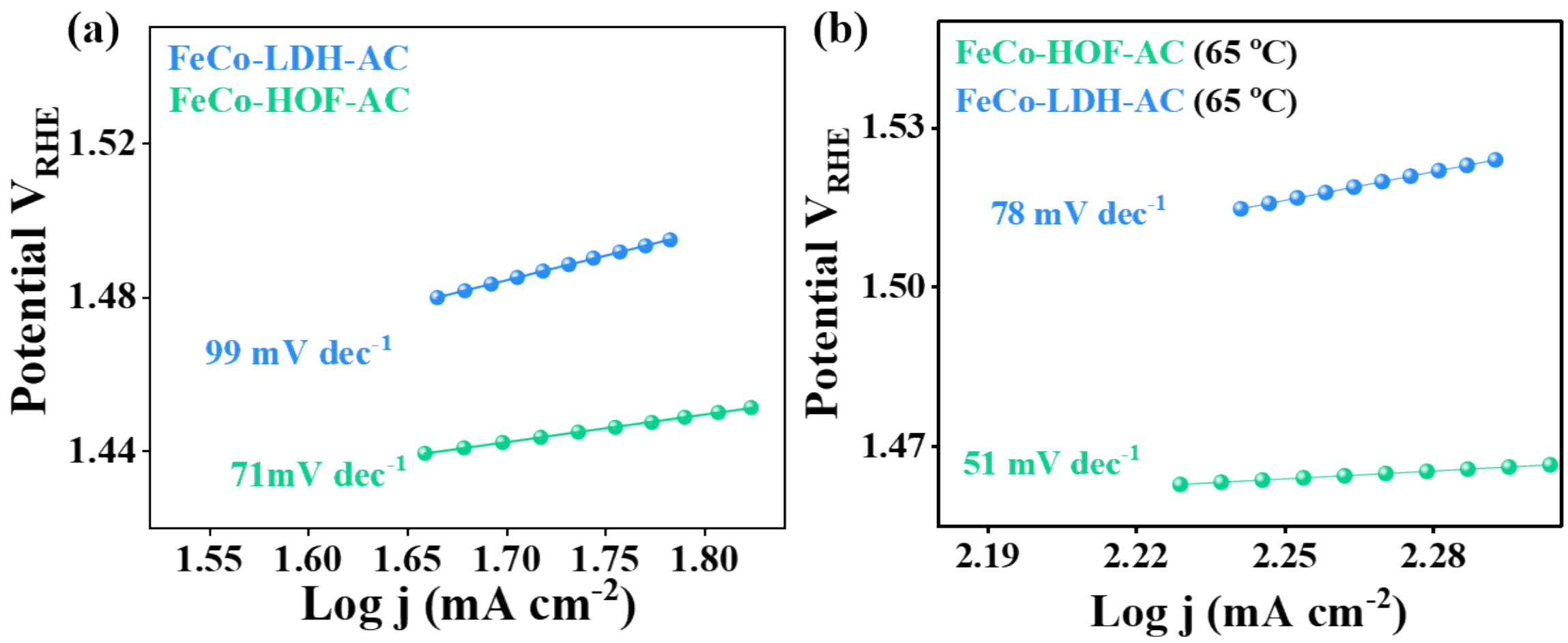

Figure S21. Tafel plots for the active catalysts. The lowest Tafel slope for FeCo-HOF-AC suggests the fastest OER kinetics

Table S3. Comparison of the $PhCH_2OH$ oxidation activity of FeCo-HOF-AC with literature reported Fe, Ni, Co-based catalysts.

Catalyst	$PhCH_2OH$ concentration	Temperature (°C)	Highest Current density (mA/cm²)	Potential (V_{RHE})	Ref.
This study					
FeCo-HOF-AC	0.1 M	65	1600	1.39	This work
FeCo-HOF-AC	0.1 M	35	910	1.53	This work
FeCo-LDH-AC	0.1 M	65	1025	1.46	This work
FeCo-LDH-AC	0.1 M	35	750	1.67	This work
Reported catalysts attained industrial current density of 400 mA cm^{-2}					
FePt-DAC	0.1 M		1500	1.49	7
Ni_3N/NM/NF	0.1 M		1200	1.47 (85 °C)	8
FeCo(O)OH/NF	0.1 M		1000	1.51	9
Co(O)OH-1/NF	0.1 M		967	1.57	10
NiOOH/NiFeOOH	0.1 M		800	1.47 (60 °C)	11
N–Mo–Ni/NF	0.1 M		700	1.43	12
Au/Co(O)OH	0.1 M		540	1.5	13
CoNi(O)OH	0.1 M		555	1.40	14
Ni/$Co(OH)_2$	0.1 M		400	1.45	15
Ni-TMA-MOF/NF	0.1 M		400	1.65	16
Other BOR catalysts					
ZnCoNiCu-HC	0.015 M		320	1.80	17
CuO@$Ni(OH)_2$	0.1 M		275	1.80	18
CoNi/P/NF	0.060 M		265	1.59	19
$Co_{0.83}Ni_{0.17}$/C	0.010 M		30	1.42	20
Co_3O_4 nanowire/NF	0.020 M		86	1.50	21
Co-Ni-LDH/NF	0.010 M		120	1.60	22
NC@$CuCo_2N_x$/CF	0.015 M		200	1.50	23
CoFe-LDH/NF	0.015 M		50	1.51	24
hp-Ni/NF	0.010 M		10	1.42	25
Ni_2P/NF	0.1 M		30	1.32	26
C@NiO/Ni_3S_2	0.2 M		85	1.42	27
$NiCo_2O_4$/NF	0.05 M		100	1.46	28

Calculation of Turn over frequency (TOF) active catalysts. [1,6]

$$TOF = (j \times N_A) / (4 \times F \times n)$$

Where,

j = current density at η = 300 mV

N_A = Avogadro number

F = Faraday constant

n = number of active Co-sites

For FeCo-HOF-AC

$TOF = [(1000 \times 10^{-3}) (6.023 \times 10^{23})] / [(96485) (4) (3.38 \times 10^{15})]$

$\mathbf{TOF = 461.71\ s^{-1}}$

For FeCo-LDH-AC

$TOF = [(72.00 \times 10^{-3}) (6.023 \times 10^{23})] / [(96485) (4) (0.78 \times 10^{15})]$

$\mathbf{TOF = 144.05\ s^{-1}}$

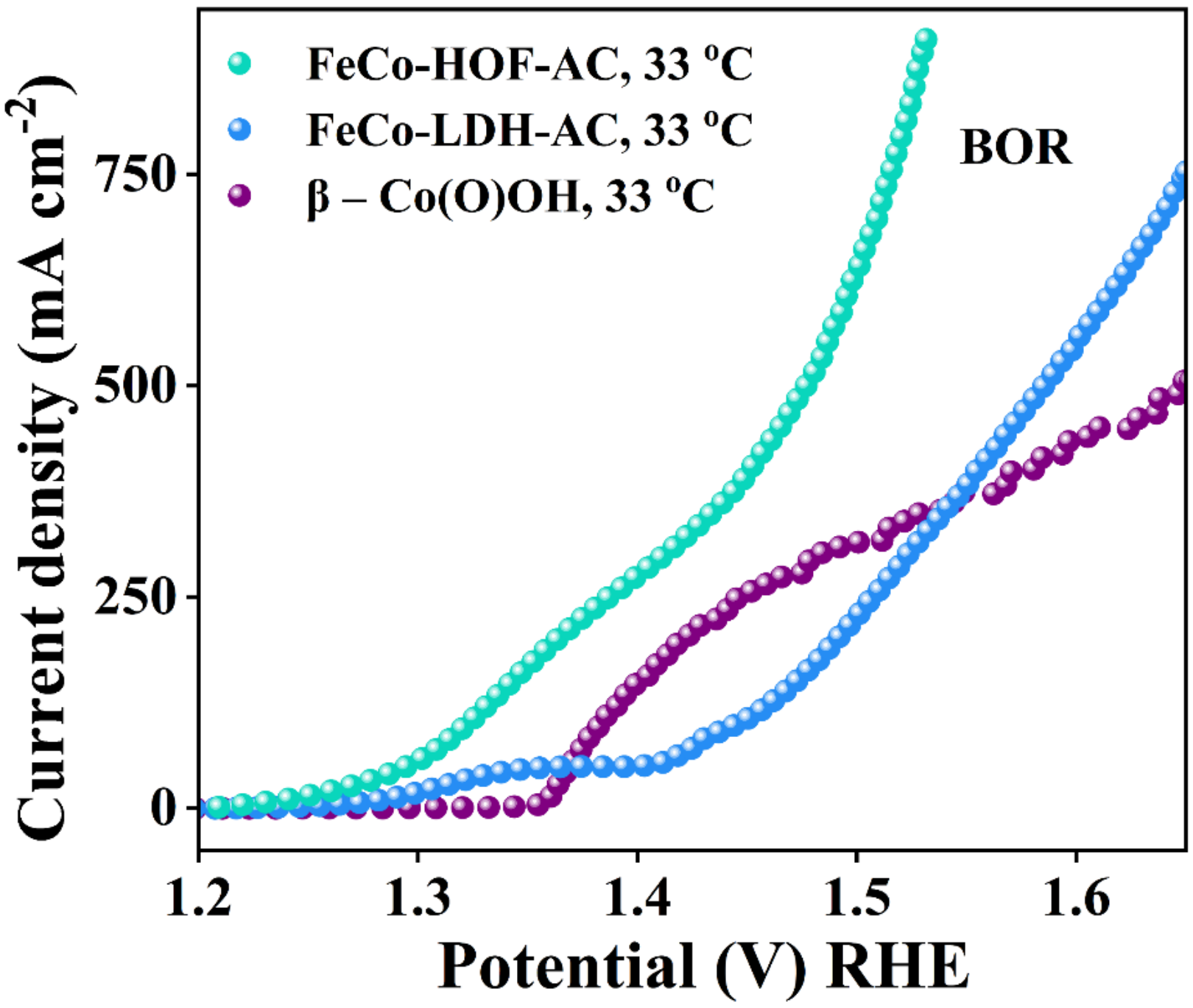

Figure S22. LSV profiles for BOR with FeCo-HOF-AC, FeCo-LDH, and β-Co(O)OH at 33 ºC.

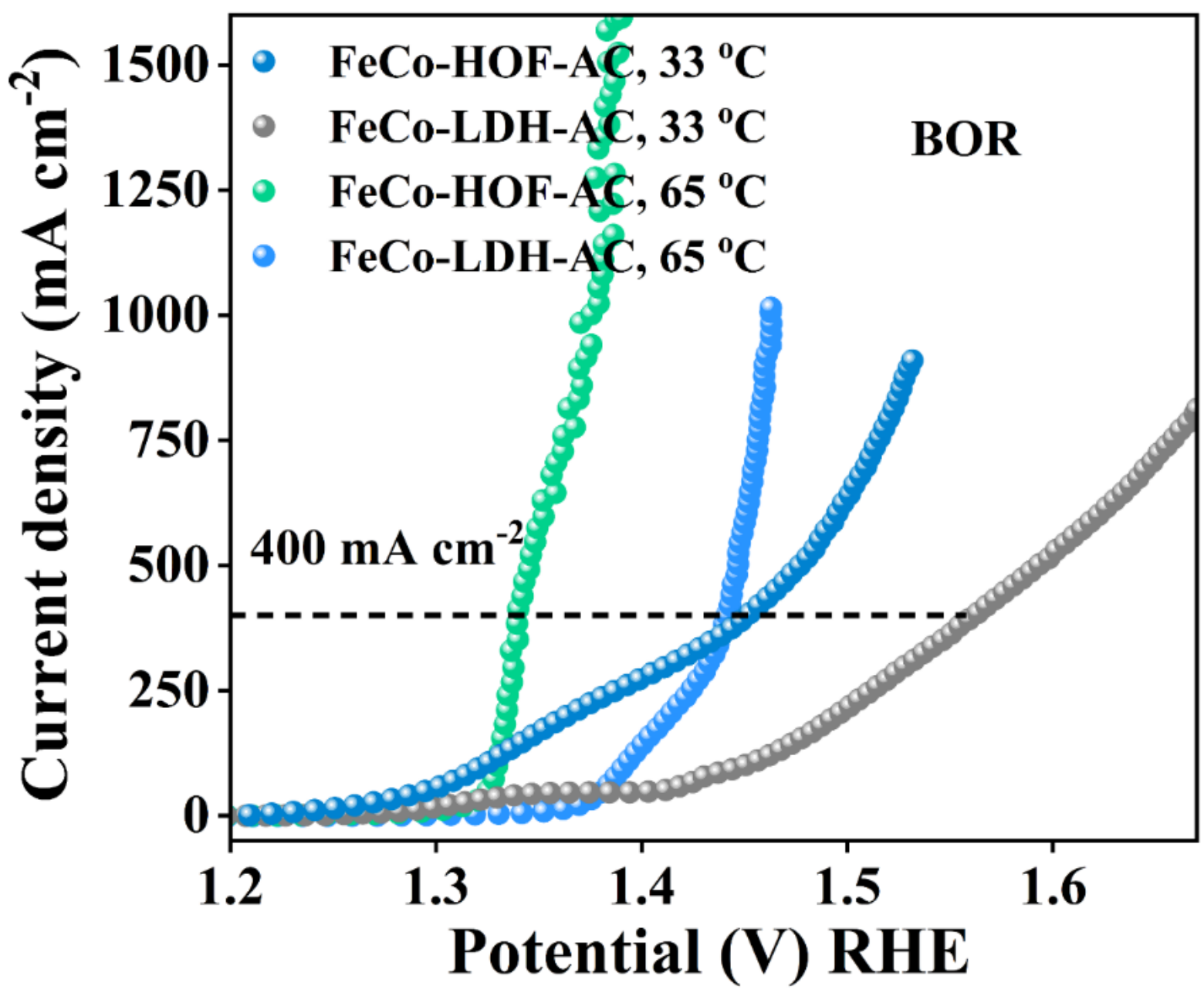

Figure S23. LSV profiles for BOR with FeCo-HOF-AC, and FeCo-LDH, at 33 ºC and 65 ºC.

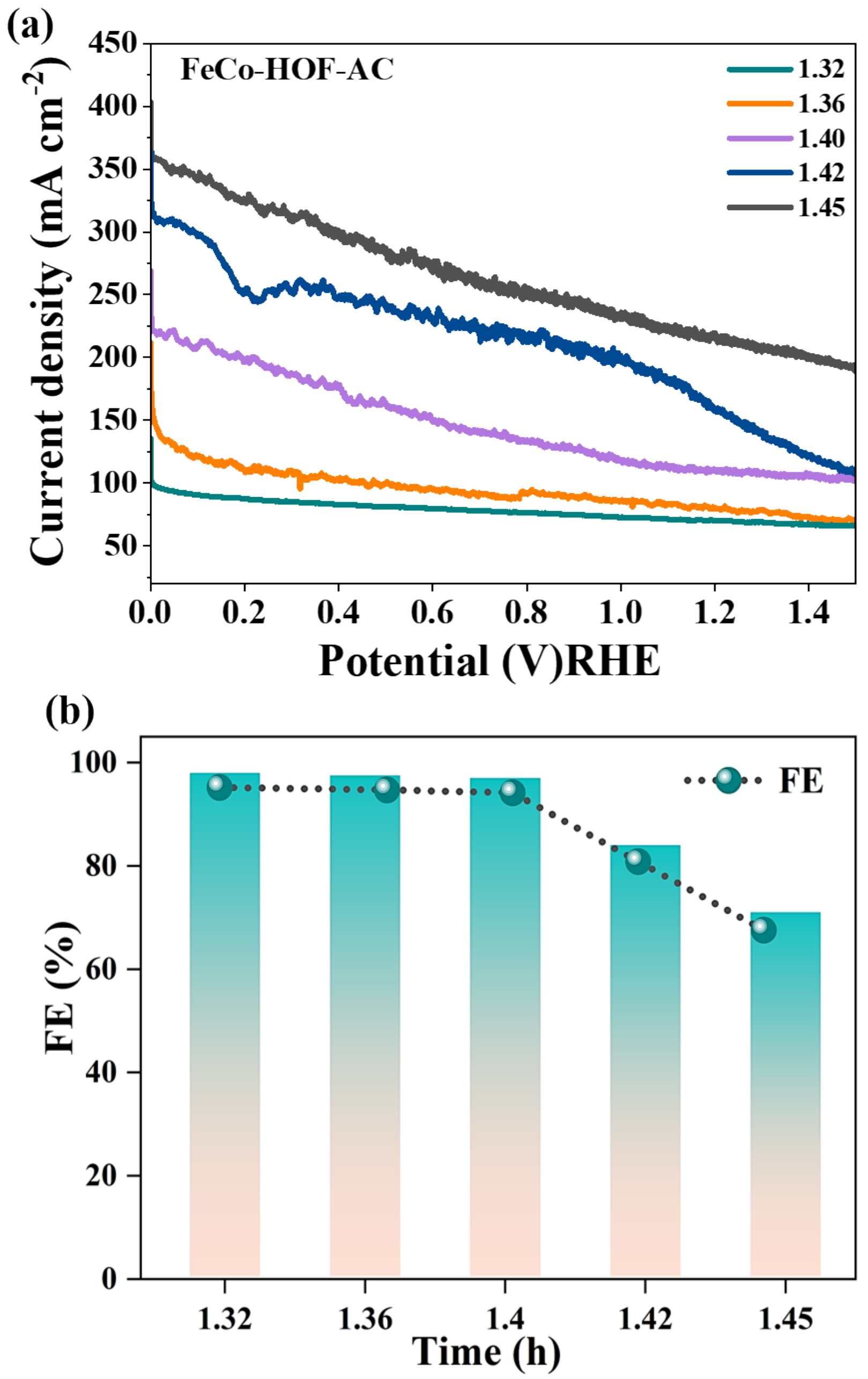

Figure S24. (a) CA study at different applied potentials for 1.5 h to optimise the benzyl alcohol oxidation activity of FeCo-HOF-AC. (b) Plot for the FE of $PhCH_2OH$ oxidation.

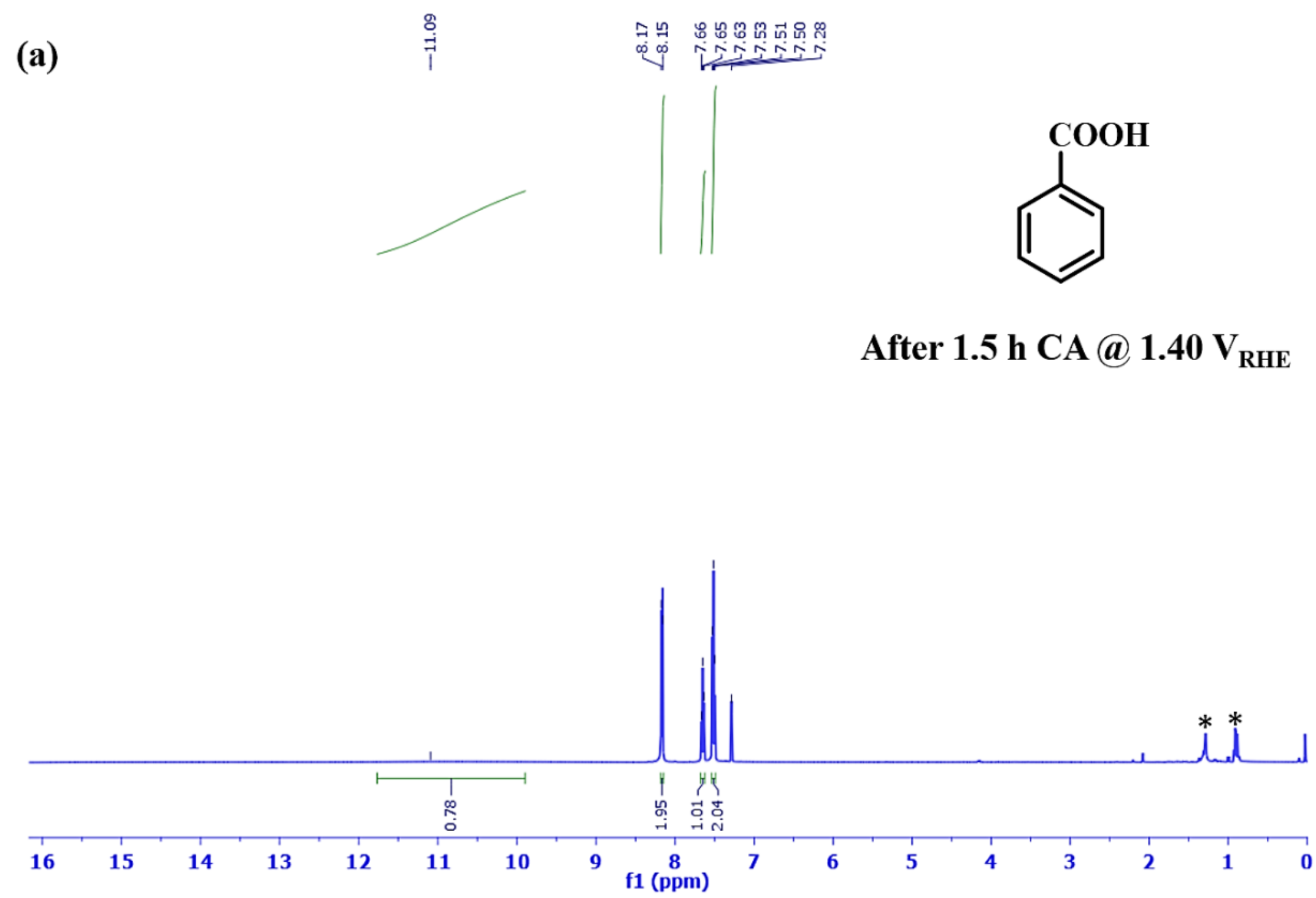

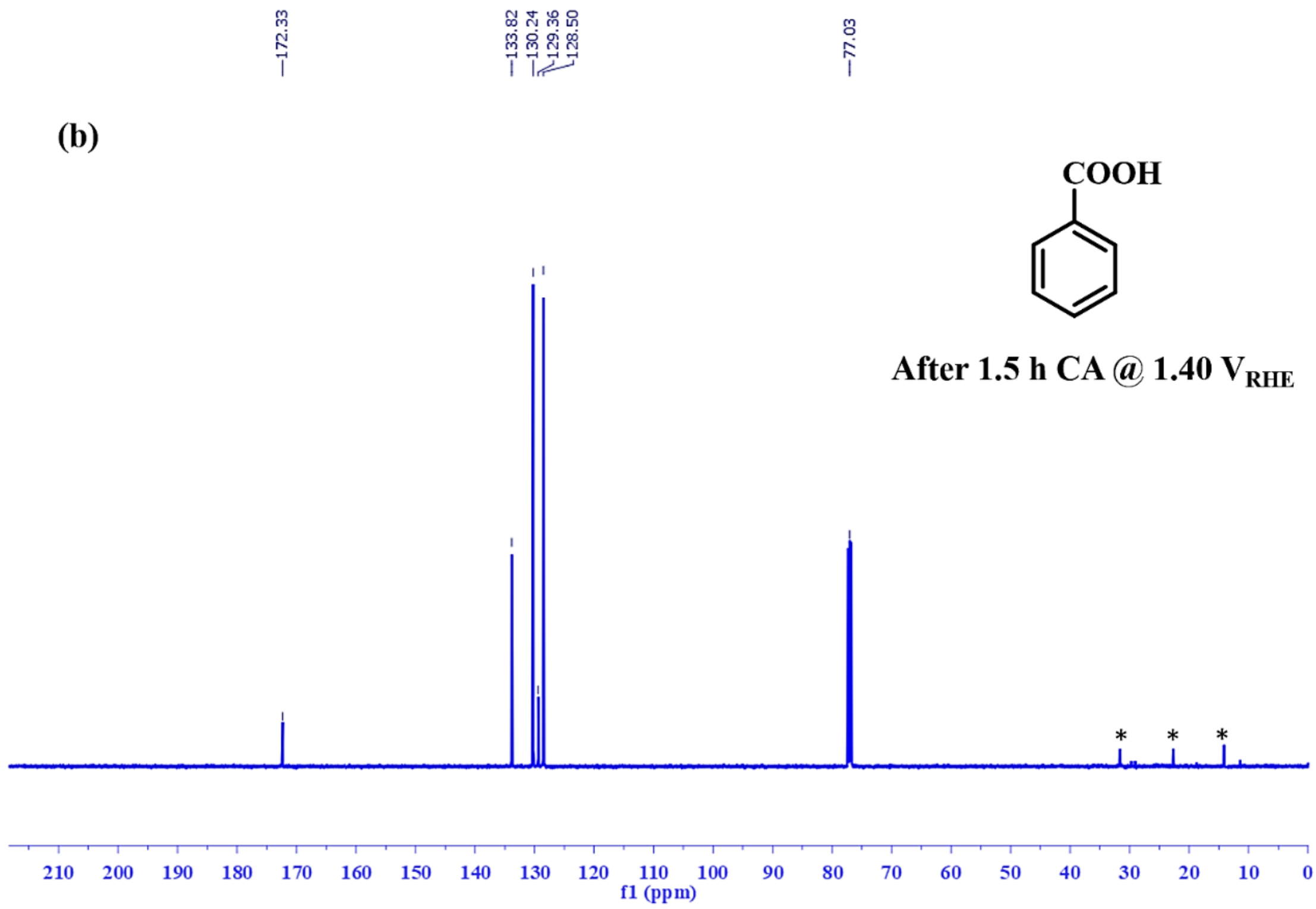

Figure S25. (a) ^{1}H NMR and (b) ^{13}C NMR spectrum of benzoic acid formed after 1.5 h of CA oxidation of 0.1 M benzyl alcohol at 1.40 V $_{RHE}$ with FeCo-HOF-AC. The * peak represents the grease impurities.[29]

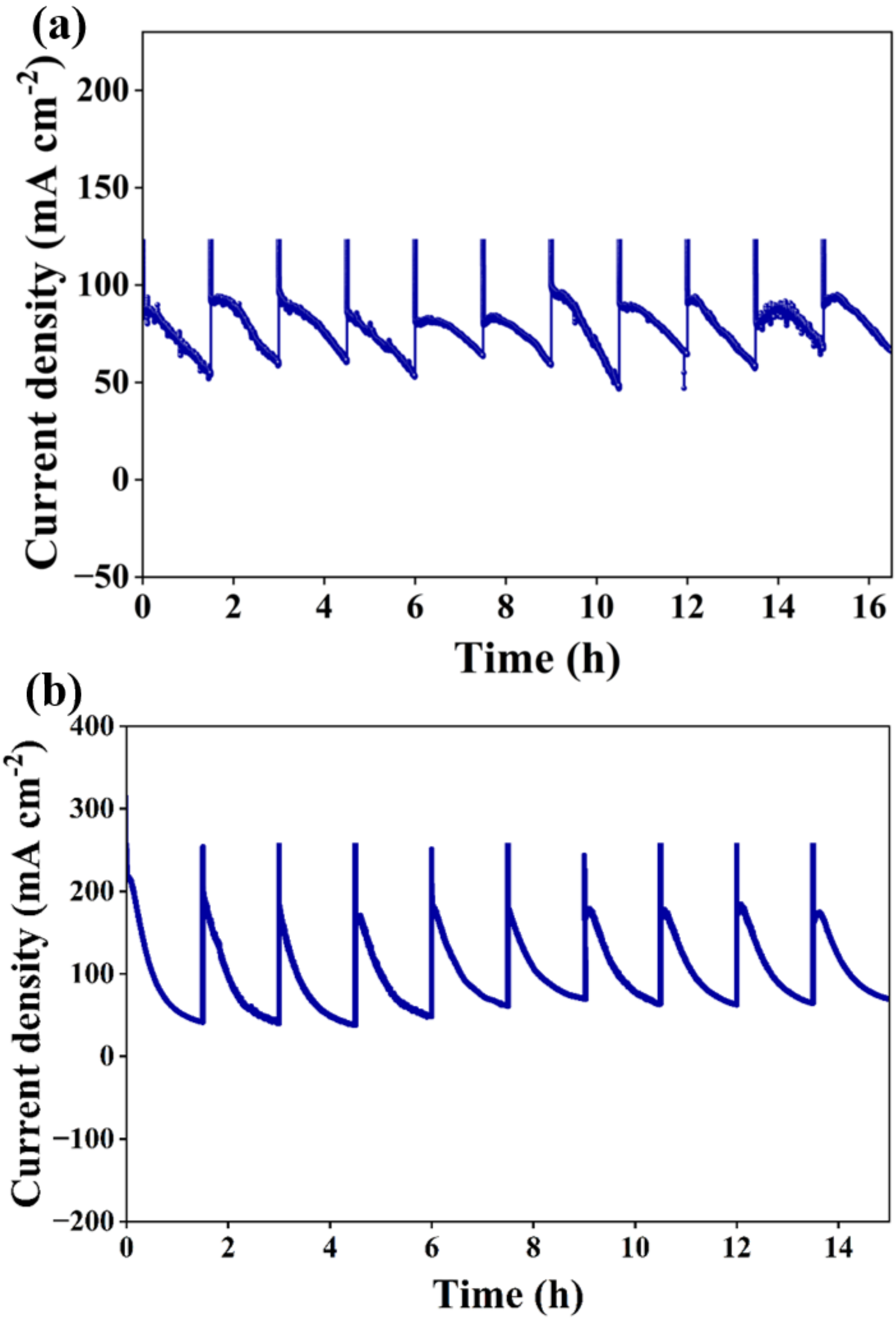

Figure S26. The chronoamperometric profile of FeCo-HOF-AC over 10 consecutive BOR cycles at 35 ºC and, (b) 65 ºC.

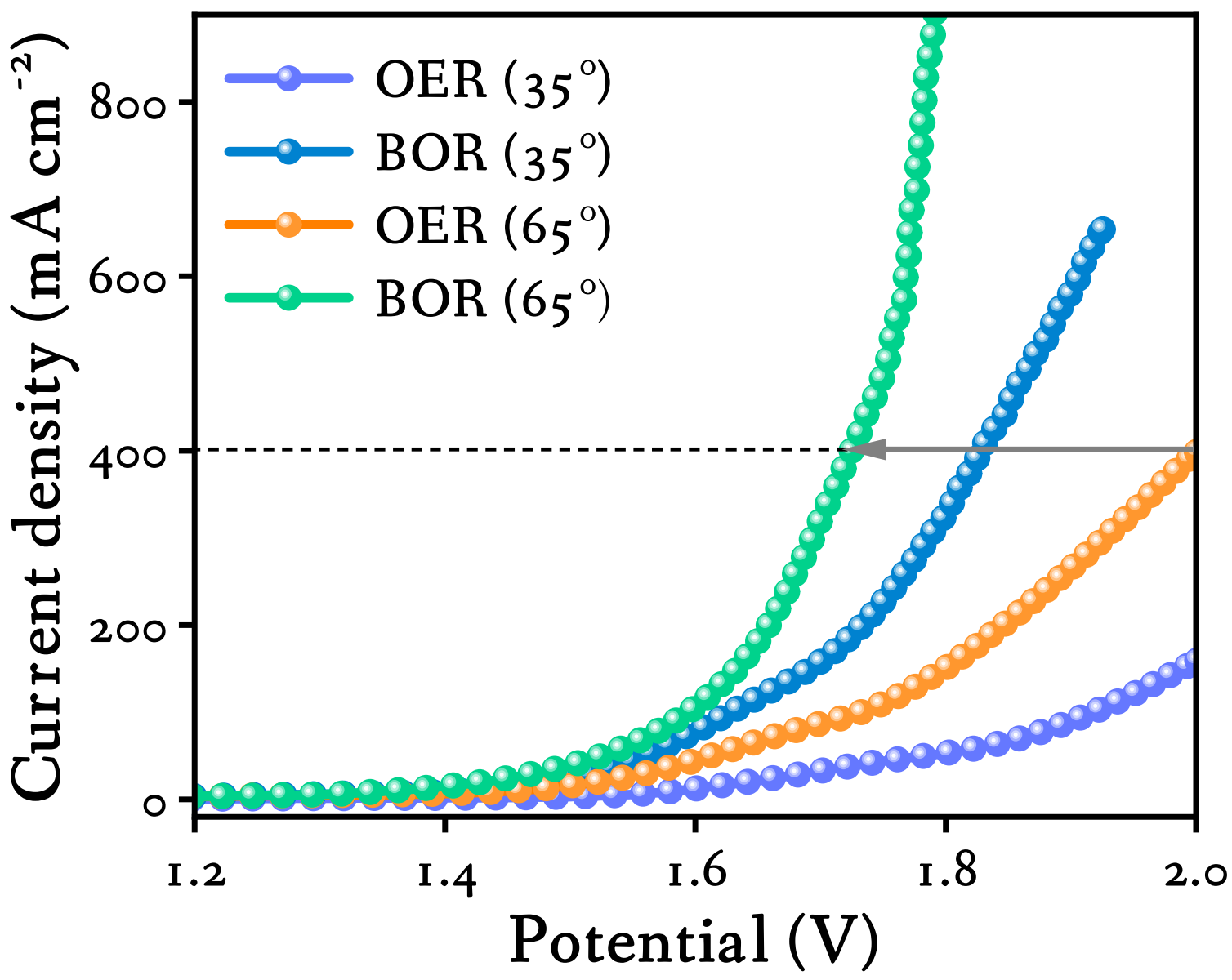

Figure S27. LSV profiles for FeCo-HOF-AC in a two-electrode system with or without BOR (Reaction conditions for BOR: 1.0 M KOH + 0.1 M $PhCH_2OH$ at a scan rate of 5 mV s^{-1}).

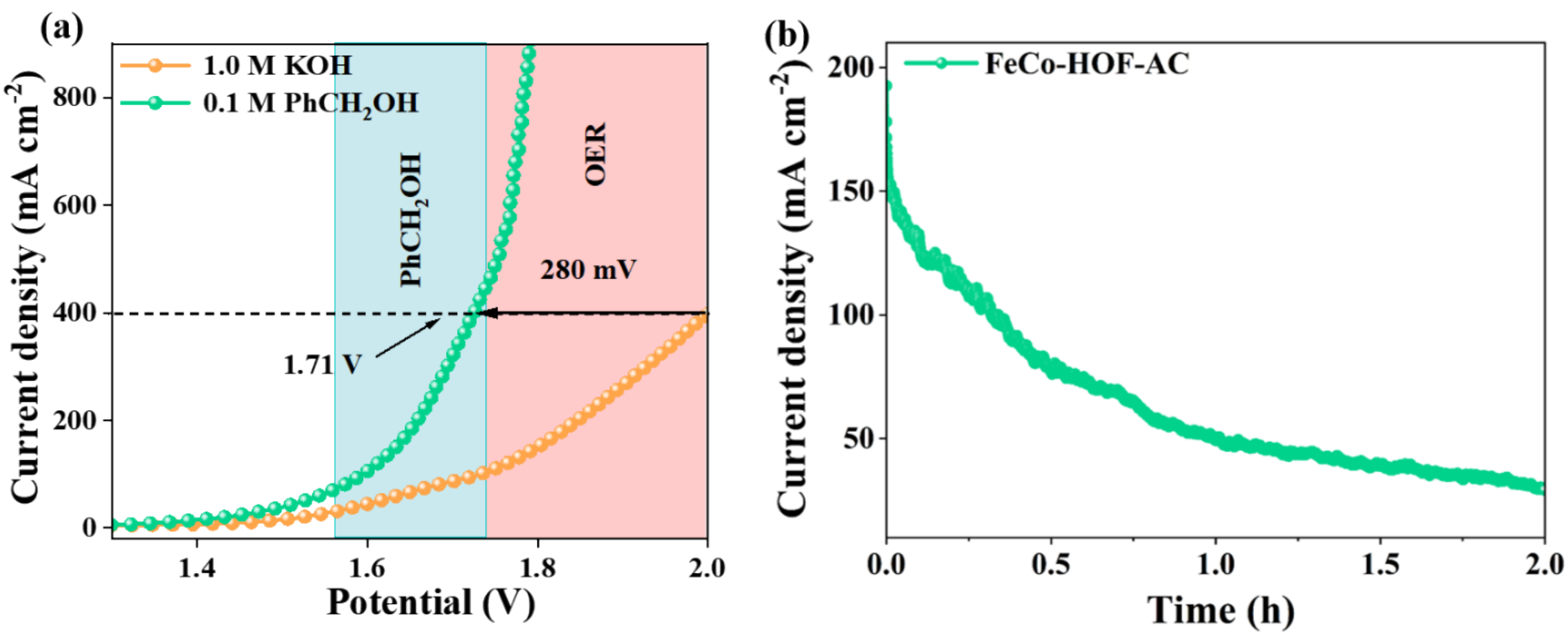

Figure S28. LSV profiles for FeCo-HOF-AC in a two-electrode system with or without BOR (Reaction conditions for BOR: 1.0 M KOH + 0.1 M $PhCH_2OH$ at a scan rate of 5 mV s^{-1}), demonstrating a 280-mV reduction in the applied potential for BOR compared to OER. (b) CA study for the benzyl alcohol oxidation of FeCo-HOF-AC at 1.68 V.

Table S4. Comparison of the $PhCH_2OH$ oxidation activity of FeCo-HOF-AC with literature reported catalyst in two-electrode configuration.

Catalyst	$PhCH_2OH$ concentration	Highest Current density (mA/cm^2)	Potential (V)	References
**FeCo-HOF-AC**	**0.1 M $PhCH_2OH$**	**905**	**1.78**	**This work**
CoNiP/NF	0.2 M $PhCH_2OH$	1900	2.35	19
FeCoNiAlMo/CNT	0.2 M $PhCH_2OH$	40	1.60	30
Ni-OH/NF	0.1 M $PhCH_2OH$	150	2.00	31
$CuO@Ni(OH)_2$	0.2 M $PhCH_2OH$	1200	3.00	18
$NiCo_2O_4$/NF	0.05 M $PhCH_2OH$	160	2.00	28
Au/CoOOH	0.2 M $PhCH_2OH$	1000	3.25	13
Cr-NF	0.1 M $PhCH_2OH$	2500	3.10	32

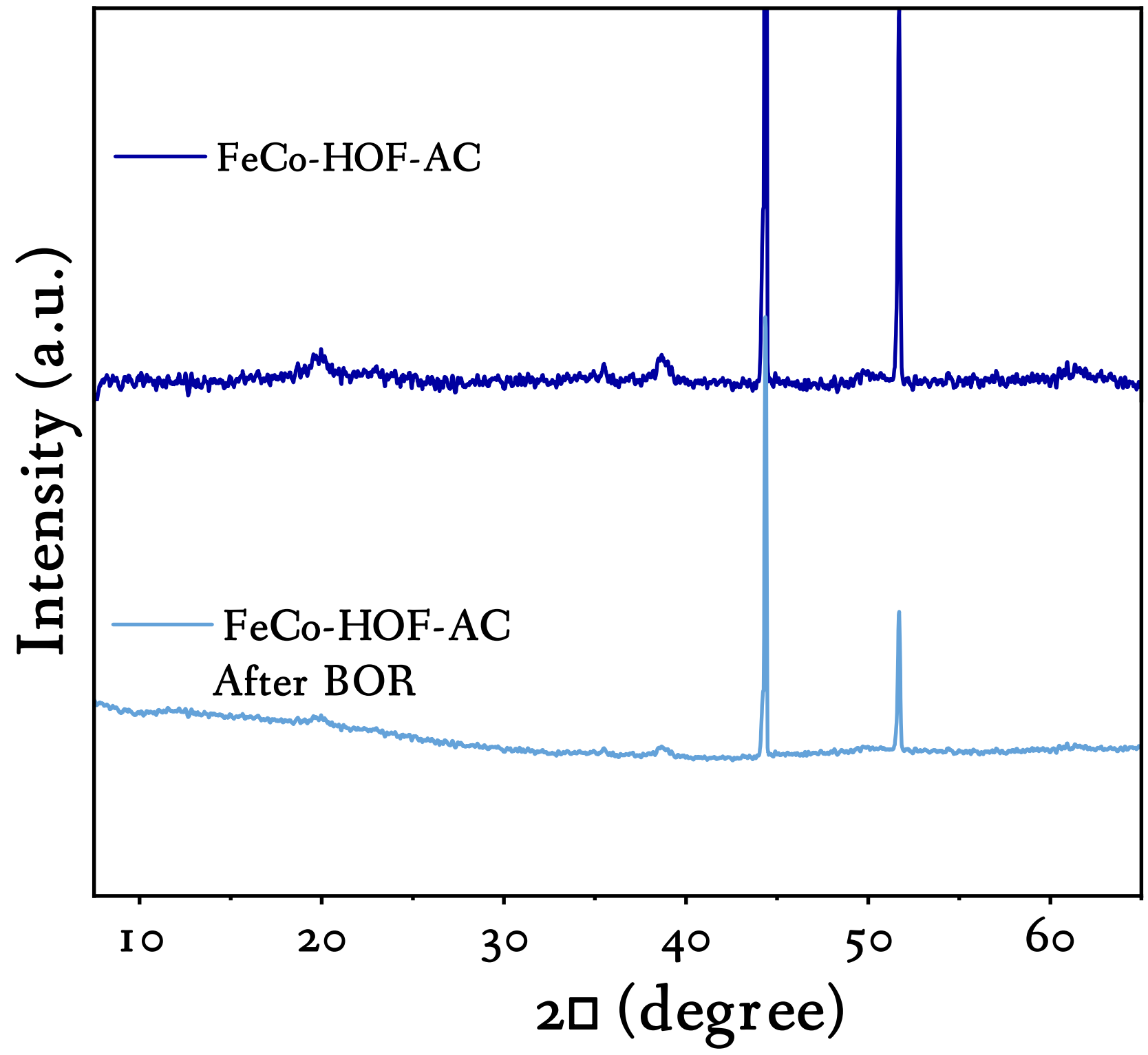

Figure S29. The PXRD pattern of FeCo-HOF-AC after $PhCH_2OH$ oxidation.

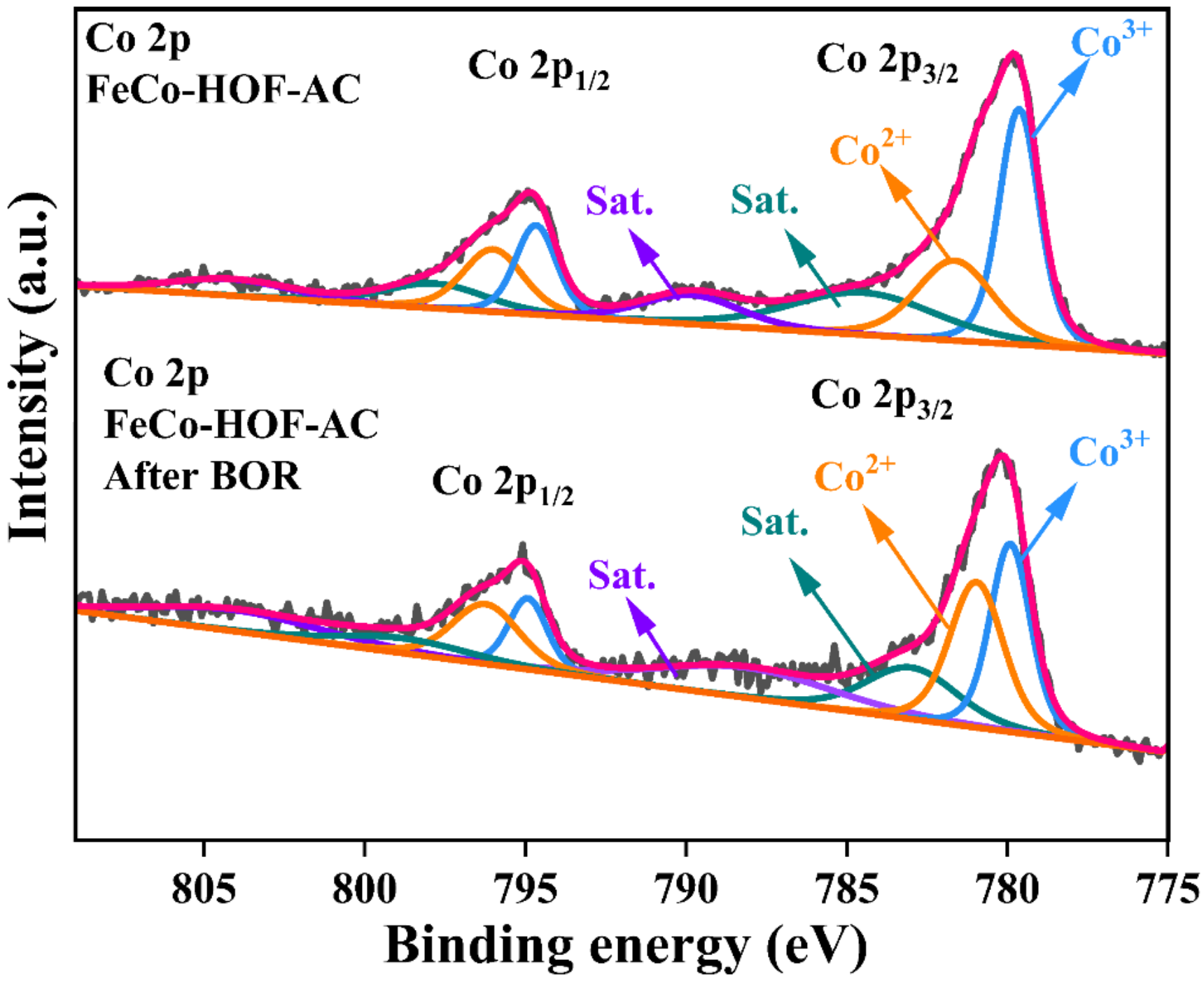

Figure S30. Co 2p XPS of FeCo-HOF-AC after $PhCH_2OH$ oxidation. The Co 2p XPS of FeCo-HOF-AC after $PhCH_2OH$ oxidation was deconvoluted into two main peaks corresponding to Co $2p_{3/2}$ and Co $2p_{1/2}$.[1,6] The Co $2p_{3/2}$ and Co $2p_{3/2}$ peaks were further split into two peaks for Co^{2+} and Co^{3+} species.

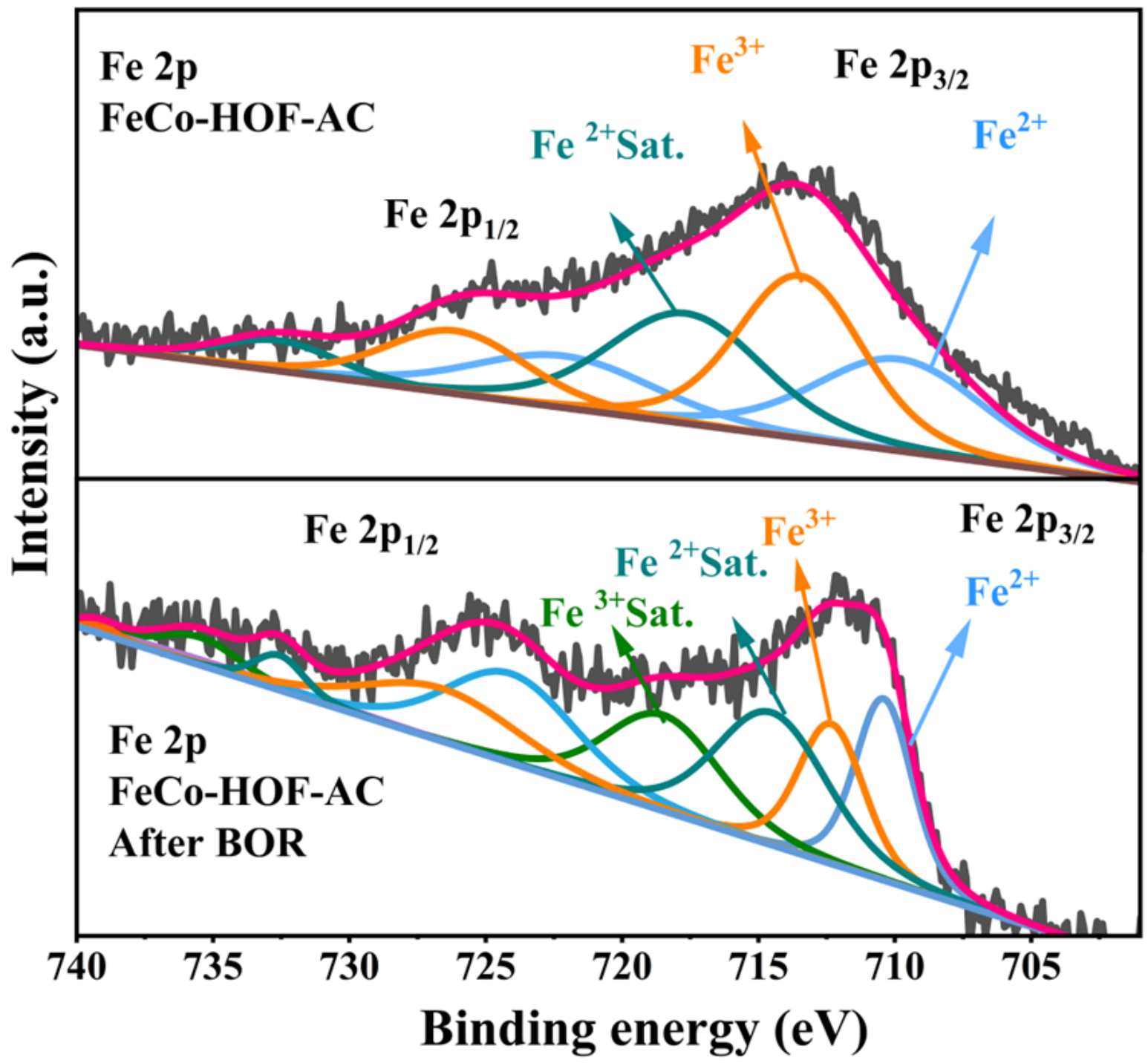

Figure S31. Fe 2p XPS of FeCo-HOF-AC after $PhCH_2OH$ oxidation. The Fe 2p XPS of FeCo-HOF-AC after $PhCH_2OH$ oxidation was deconvoluted into two main peaks corresponding to Fe $2p_{3/2}$ and Fe $2p_{1}/2$.[1,6] The Fe $2p_{3/2}$ region was further split into two peaks for Fe^{2+} and Fe^{3+} species.[1,6]

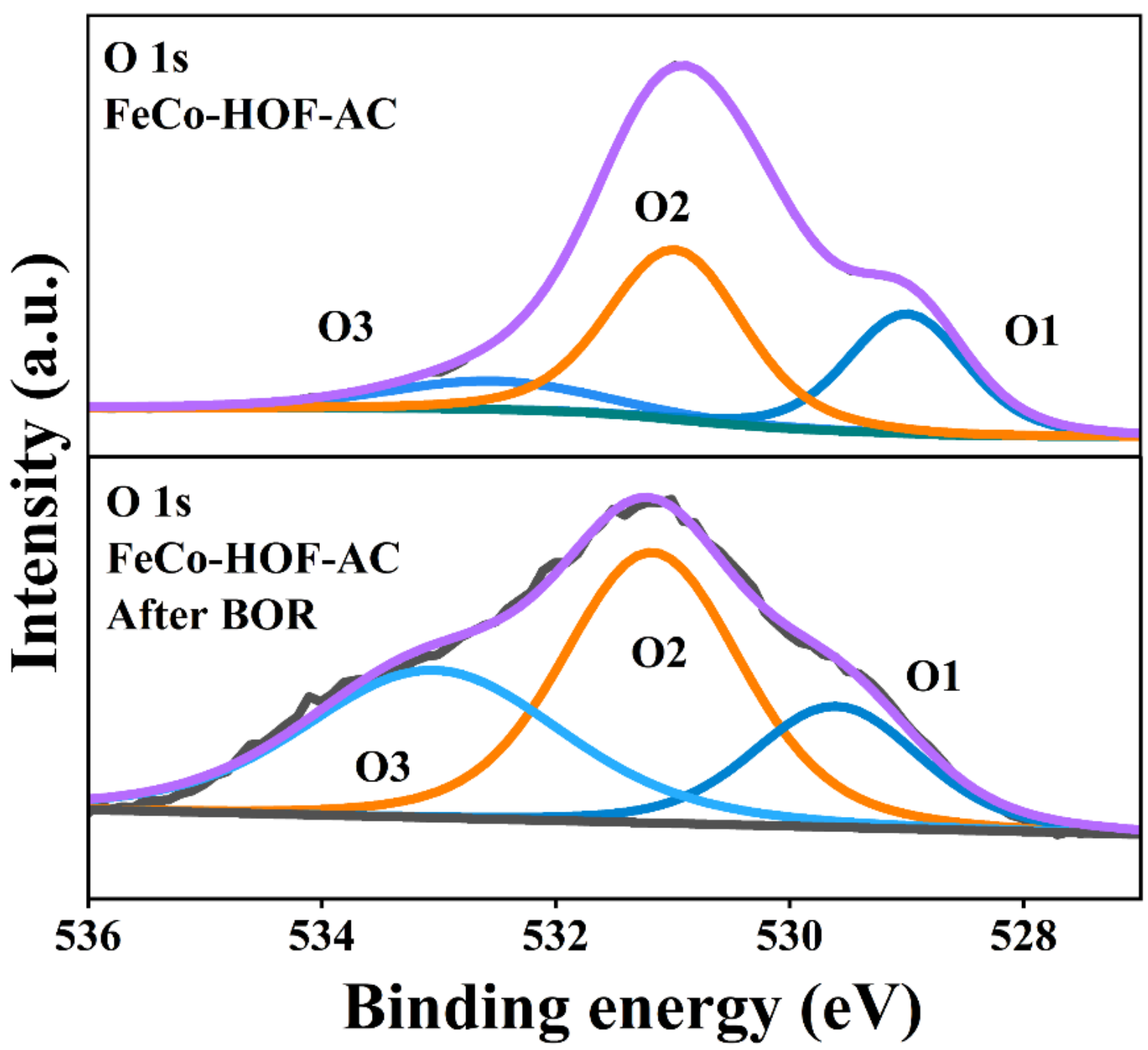

Figure S32. O 1s XPS of FeCo-HOF-AC after anodic activation and BOR. After the anodic activation and BOR, the XP spectrum can be deconvoluted into three different oxygen species: O1 lattice oxygen, O2 –OH group, and O3 absorbed water molecule.[1,6]

References

1 J. Huang, Z. Zhang, C. Spezzati, A. H. Clark, N. Hales, N. S. Genz, N. Daffé, R. Skoupy, L. Gubler, I. E. Castelli, T. J. Schmidt and E. Fabbri, *Nat. Commun.*, 2025, **16**, 7518.

2 A. Yadav, T. Ansari, P. Mannu, B. Singh, A.K. Singh, Y.C. Huang, V. Kumar, S. Singh, C.L. Dong and A. Indra. *J. Mater. Chem. A* 2024, **12** 29072–29080.

3 K. V. Nielson, L. Zhang, Q. Zhang and T. L. Liu. *Inorg. Chem.,* 2019, **58**, 10756–10760.

4 M. E. Ziebel, J.C. Ondry and J. R. Long. *Chem. Sci.*, 2020, **11**, 6690–6700.

5 Y. Chen, Q. Li, Y. Lin, J. Liu, J. Pan, J. Hu and X. Xu. *Nat. Commun.,* 2024, **15**, 7278.

6 B. Singh, P. Mannu, Y.C. Huang, R. Prakash, S. Shen, C. L. Dong and A. Indra. *Angew. Chem. Int. Ed.*, 2022, **61** 202211585.

7 G. Song, W. Sun, X. Wang, S. Gong and Y. Tian. *Nano Res.*, 2025, **18**, 94907362.

8 S. Ghosh, J.N. Hausmann, L. Reith, G. Vijaykumar, J. Schmidt, K. Laun, S. Berendts, I. Zebger, M. Driess and P.W. Menezes. *Adv. Energy Mater.*, 2024, **14**, 2400356.

9 B. Singh, T. Ansari, N. Verma, Y.C. Huang, P. Mannu, C. L. Dong and A. Indra. *J. Mater. Chem., A* 2024, **12,** 19321–19330.

10 V. Kumar, V. Patel and A. Indra. *J. Mater. Chem. A* 2026, **14**, 16424–16431.

11 I. Mondal, P. V. Menezes, K. Laun, T. Diemant, M. Al-Shakran, I. Zebger, T. Jacob, M. Driess and P. W. Menezes, *ACS Nano* 2023*,* **17**, 14043–14052.

12 J. Wan, X. Mu, Y. Jin, J. Zhu, Y. Xiong, T. Li and R. Li. *Green Chem.,* 2022, **24**, 4870–4876.

13 Z. Li, Y. Yan, S. M. Xu, H. Zhou, M. Xu, L. Ma, M. Shao, X. Kong, B. Wang, L. Zheng and H. Duan. *Nat. Commun.*, 2022, **13**, 147.

14 T. Ansari, D. Bagchi, S. Ghosh, J. Niklas Hausmann, A. Indra and P. W. Menezes. *Chem. Eur. J.*, 2025, **31**, e202404174.

15 H. Huang, C. Yu, X. Han, H. Huang, Q. Wei, W. Guo, Z. Wang and J. Qiu. *Energy Environ. Sci.*, 2020, **13**, 4990–4999.

16 W. Su, X. Zheng, W. Xiong, Y. Ouyang, Z. Zhang, W. Zeng, H. Duan, X. Chen, P. Su, Z. Sun and M. Yuan. *Inorg. Chem.*, 2024, **63**, 12572–12581.

17 S. Wang, L. Gao, L. Sun, H. Zhan, Z. Han, Z. Wang, J. Xie and X. Sun. *Chem. Eur. J.*, 2026, **32**, 70798.

18 X. Du, W. Xie, Y. Wang, H. Li, J. Li, Y. Li, Y. Song, Z. Li, J. Y. Lee and M. Shao. *AIChE J.* 2024*,* **70**, 18469.

19 S. Guo, L. Ren, S. Jia, S. Zhi, J. Chang, X. Ma and Z. Gao. *J. Colloid Interface Sci.*, 2026, **714** 140180.

20 G. Liu, X. Zhang, C. Zhao, Q. Xiong, W. Gong, G. Wang, Y. Zhang, H. Zhang and H. Zhao. *New J. Chem.*, 2018, **42**, 6381–6388.

21 Y. Cao, D. Zhang, X. Kong, F. Zhang and X. Lei. *J. Mater. Sci.,* 2021, **56**, 6689–6703.

22 N. Shilpa, A. Pandikassala, P. Krishnaraj, P.S. Walko, R.N. Devi and S. Kurungot, *ACS Appl. Mater. Interfaces* 2022, **14**,16222–16232.

23 J. Zheng, X. Chen, X. Zhong, S. Li, T. Liu, G. Zhuang, X. Li, S. Deng, D. Mei and J. G. Wang, *Adv. Funct. Mater.,* 2017, **27,** 1704169.

24 Y. Xu, H. Liu, Y. Wu, Q. Wu, C. Li, X. Wang, H. Qin, A. Qin and L. Wang. *ChemNanoMat* 2023, **9**, 202300414.

25 B. You, X. Liu, X. Liu and Y. Sun. *ACS Catal.*, 2017, **7**, 4564–4570.

26 L. Ming, X. Y. Wu, S. S. Wang, W. Wu and C. Z. Lu. *Green Chem.,* 2021, **23**, 7825–7830.

27 R. Li, P. Kuang, L. Wang, H. Tang and J. Yu. *Chem. Eng. J.*, 2022, **431**, 134137.

28 M. Xu, J. Geng, H. Xu, S. Zhang and H. Zhang, *Inorg. Chem. Front.*, 2023, **10**, 2053–2059.

29 H. E. Gottlieb, V. Kotlyar and A. Nudelman. *J. Org. Chem.,* 1997, **62**, 7512–7515.

30 Y. Wang, M. Ni, W. Yan, C. Zhu, D. Jiang, Y. Yuan, and H. Du. *Adv. Funct. Mater.,* 2024, **34**, 2311611.

31 L. Ming, X.Y. Wu, Sa-Sa Wang, W. Wu and C.Z. Lu, *Green Chem.,* 2021, **23**, 7825–7830.

32 D. Bhutani, S. Dutta, A. Garg, S. K Pati, and E. Muthusamy, *Small* 2025, **21**, 2500084.
